# Antiobesity Pharmacotherapy for Patients With Genetic Obesity Due to Defects in the Leptin-Melanocortin Pathway

**DOI:** 10.1210/endrev/bnaf004

**Published:** 2025-02-11

**Authors:** Mila S Welling, Elisabeth F C van Rossum, Erica L T van den Akker

**Affiliations:** Obesity Center CGG, Erasmus MC, University Medical Center Rotterdam, Rotterdam 3015 GD, The Netherlands; Department of Internal Medicine, Division of Endocrinology, Erasmus MC, University Medical Center Rotterdam, Rotterdam 3015 GD, The Netherlands; Department of Pediatrics, Division of Endocrinology, Erasmus MC-Sophia Children's Hospital, University of Medical Center Rotterdam, Rotterdam 3015 GD, The Netherlands; Obesity Center CGG, Erasmus MC, University Medical Center Rotterdam, Rotterdam 3015 GD, The Netherlands; Department of Internal Medicine, Division of Endocrinology, Erasmus MC, University Medical Center Rotterdam, Rotterdam 3015 GD, The Netherlands; Obesity Center CGG, Erasmus MC, University Medical Center Rotterdam, Rotterdam 3015 GD, The Netherlands; Department of Pediatrics, Division of Endocrinology, Erasmus MC-Sophia Children's Hospital, University of Medical Center Rotterdam, Rotterdam 3015 GD, The Netherlands

**Keywords:** obesity, genetics, targeted, non-targeted, anti-obesity medication, AOM, gene therapy

## Abstract

Lifestyle interventions are the cornerstone of obesity treatment. However, insufficient long-term effects are observed in patients with genetic obesity disorders, as their hyperphagia remains untreated. Hence, patients with genetic obesity often require additional pharmacotherapy to effectively manage and treat their hyperphagia and obesity. Recent advancements in antiobesity pharmacotherapy have expanded the range of available antiobesity medications (AOM). This includes the targeted AOM setmelanotide, approved for specific genetic obesity disorders, as well as nontargeted AOMs such as naltrexone-bupropion and glucagon-like peptide-1 analogues. Targeted AOMs have demonstrated significant weight loss, reduced obesity-related comorbidities, and improved hyperphagia and quality of life in patients with specific genetic obesity disorders. Small observational studies have shown that similar benefits from nontargeted AOMs or off-label pharmacotherapies can be achieved in patients with specific genetic obesity disorders, compared to common multifactorial obesity. In the future, novel and innovative pharmacotherapeutical options, including combination therapies and possibly gene therapy, will emerge, offering promising effects on body weight, hyperphagia, and, most importantly, quality of life for patients with a variety of genetic obesity disorders.

## Graphical Abstract

**Graphical Abstract:**
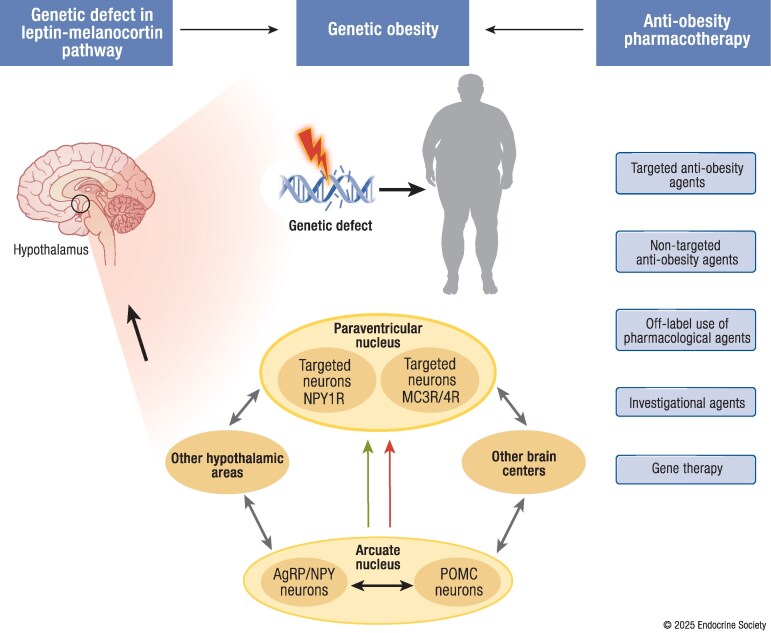

**Essential points**

- Patients with genetic obesity disorders often require treatment with antiobesity pharmacotherapy, in addition to lifestyle interventions, to effectively manage their obesity and hyperphagia.
- Recent advancements in antiobesity pharmacotherapy have expanded the range of available antiobesity medications (AOM), including both targeted and nontargeted agents.
- The targeted AOM, setmelanotide, is approved only for specific genetic obesity disorders and has demonstrated beneficial effects on weight, quality of life, and hyperphagia.
- The availability of targeted agents underscores the importance of genetic testing in patients suspected for genetic obesity disorders.
- Nontargeted AOMs, such as naltrexone-bupropion, glucagon-like peptide-1 analogues, and other off-label agents, show similar benefits in obesity treatment compared to common multifactorial obesity in small observational studies.
- In the future, novel and innovative pharmacotherapeutical options, including combination therapies and possibly gene therapy, will emerge, offering promising effects on weight, hyperphagia, and, most importantly, quality of life for patients with genetic obesity disorders.

As obesity is associated with numerous comorbidities, such as cardiovascular and metabolic diseases, depression, joint problems, infertility, and various types of cancer, reducing body weight and treating obesity is of paramount importance (1). Without efficient prevention and treatment, the impact of obesity on morbidity and mortality is enormous. Consequently, obesity has a substantial economic impact worldwide, including high direct and indirect costs, such as loss of productivity at work (2, 3).

The most common cause of obesity is a complex interplay of biological factors, including (epi)genetic predisposition, and environmental factors, such as increasing sedentary behaviors, reduced physical activity, and an overabundance of food resulting in (over)consumption. Additionally, other contributors often play a role, such as psychosocial and sociocultural factors and use of weight-inducing medications (4). Consequently, this is often referred to as common (multifactorial) obesity (5). However, possible underlying medical causes of obesity can be present as well, such as endocrine diseases, hypothalamic dysfunction, and genetic obesity disorders (4). These genetic obesity disorders were previously assumed to be extremely rare. However, a UK birth cohort study reported a prevalence of 0.3% of only melanocortin-4 receptors loss of function mutations (6). Additionally, this prevalence could increase up to 13% in a priori suspected population, as shown in a study including children with severe obesity referred to a tertiary obesity center because of a suspicion of an underlying medical cause of obesity (7). Also in adults, the prevalence appears to be less rare than previously thought. In our Dutch study, we identified a definite genetic obesity disorder in 3.9% of predominantly adult patients referred to a medical center specialized in obesity, and a potential diagnosis in an additional 5.4% of cases (8). Even with increasing knowledge on genetic obesity and obesity-related genes and the development of modern and fast sequencing techniques, genetic obesity disorders are still often unrecognized (9).

Genetic obesity disorders are brain diseases that result in the disruption of the neuroendocrine system of the hypothalamus. They often arise due to genetic defects in the leptin-melanocortin pathway (10). This hypothalamic pathway, is essential for the homeostatic regulation of body weight (Fig. 1). In short and simplified, leptin, derived from the peripheral adipose tissue, binds to the leptin receptor (LEPR) in the hypothalamus. Activation of LEPR by leptin, which is promoted by Src homology 2B adaptor protein (SH2B1), subsequently leads to production of pro-opiomelanocortin (POMC), which on its turn is cleaved by the proprotein convertase 1 (encoded by proprotein convertase subtilisin and kexin type 1 [PCSK1]) into α-melanocyte-stimulating hormone (α-MSH) and β-MSH. Finally, α-MSH binds to the melanocortin-4 receptor (MC4R), resulting in increased satiety and reduced food intake (11). Other factors, like neural or endocrine signals, such as ghrelin, peptide YY, and cholecystokinin, from the gastrointestinal tract, can influence appetite as well (12). A defect in any of the genes involved in the leptin-melanocortin pathway, consequently, can result in less functional or defective protein products, leading to impaired signaling. Examples of affected genes, resulting in either syndromic or nonsyndromic genetic obesity, are the previously mentioned *LEPR*, *SH2B1*, *POMC*, *PCSK1*, or *MC4R* genes, as well as numerous other genes, such as *NCOA1*, *SIM1*, and *PHIP*, involved in the leptin-melanocortin pathway. Ciliopathies, like Bardet-Biedl syndrome (BBS) and Alström syndrome (AS), are also part of the genetic obesity disorder spectrum. In addition to the role of the hypothalamic leptin-melanocortin pathway, several studies using genome-wide association study data have shown associations between gene variants, associated with energy metabolism and adipocyte biology, with body mass index (BMI) and obesity (13, 14).

**Figure 1.**
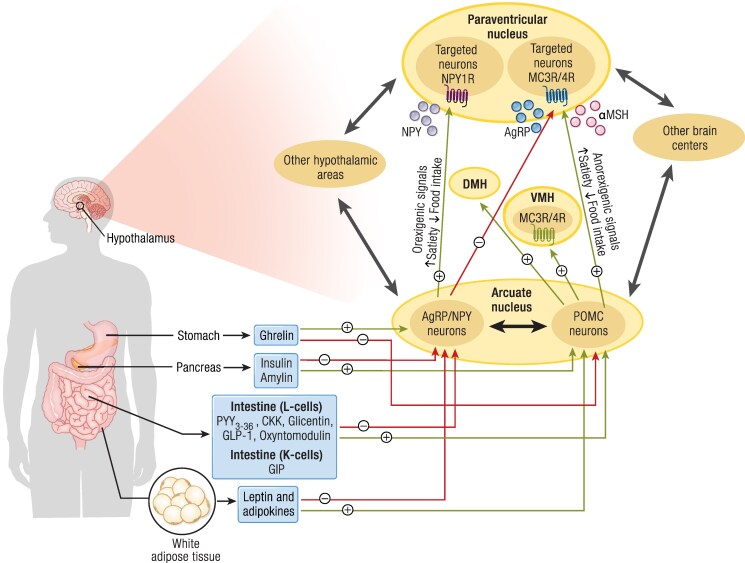
The figure provides an overview of the topic of this review. Genetic defects in the leptin-melanocortin pathway, located within the hypothalamus, play a crucial role in regulating energy balance, appetite, and behavior through 2 opposing types of first-order neurons in the arcuate nucleus: orexigenic agouti-related peptide/neuropeptide Y (AgRP/NPY) neurons and anorexigenic pro-opiomelanocortin (POMC) neurons. These hypothalamic neurocircuits connect with other brain regions to control both the physiological and hedonic aspects of food intake, influenced by afferent signals from gastrointestinal and adipose tissues. POMC neurons act through melanocortin-3 and 4 receptors (MC3R and MC4R) via α-melanocyte-stimulating hormone, an agonist that promotes anorexigenic effects, while AgRP/NPY serves as an inverse agonist, opposing these effects. NPY, produced by AgRP/NPY neurons, further promotes food intake and suppresses energy use. Both neuron types signal to the paraventricular nucleus of the hypothalamus, a key second-order site expressing MC4R and NPY1R receptors. The paraventricular nucleus integrates inputs to modulate appetite and energy homeostasis. Genetic defects in this leptin-melanocortin pathway lead to the clinical picture of genetic obesity with hyperphagia and severe early-onset obesity. Abbreviations: α-MSH, alpha-melanocyte-stimulating hormone; CCK, cholecystokinin; DMH, dorsomedial nucleus of the hypothalamus; GIP, glucose-dependent insulinotropic polypeptide; GLP-1, glucagon-like peptide-1; PYY, peptide YY; VMH, ventromedial nucleus of the hypothalamus. Adapted from Sweeney et al. *Nat Rev Endocrinol,* 2023;19(9):507-519. © Springer Nature Limited. And reprinted with permission from Angelidi et al. *Endocr Rev,* 2022;43(3):507-557. © The Authors. Published by Oxford University Press on behalf of the Endocrine Society.

These genetic defects often lead to the distinctive phenotypic features of early-onset obesity and hyperphagia (ie, insatiable hunger, decreased satiation and satiety, and preoccupation with food). Especially, this hyperphagia poses these patients at tremendous psychological stress and negatively affects their quality of life if being untreated (15, 16). Moreover, the early onset of obesity predisposes these patients at additional risk compared to patients with common obesity, as the duration of obesity is significantly longer in these patients. For example, type 2 diabetes and atherosclerosis have been associated with the duration of obesity (17). This highlights the importance of early diagnosing of genetic obesity, effective prevention, and, in case of obesity, efficient treatment to reduce body weight, leading to reduced risks of obesity-related comorbidities and negative health sequelae. Currently, the Endocrine Society has proposed recommendations only on to perform genetic testing in children with obesity (18). A recent study showed that these recommendations have low sensitivity for detecting genetic obesity in adults, indicating the need for further research to develop recommendations for genetic testing in adults with obesity as well. The study also demonstrated a significant younger age of onset of obesity and higher prevalence of impaired appetite regulation in adults with genetic obesity compared to patients with common multifactorial obesity (19).

Lifestyle interventions, focusing on diet and physical activity, is the cornerstone of obesity treatment in general. In some cases, cognitive behavioral therapy is added. These lifestyle treatments are also advised for patients with genetic obesity, adjusted to the specific needs of the patient with genetic obesity. For example, cognitive behavioral therapy to learn how to cope with the hyperphagia may be valuable for both adults and children with genetic obesity. In addition, children with genetic obesity and severe hyperphagia and their families often need a multidisciplinary team, including a pediatrician, dietician, physical therapist, and family therapist, to guide them and give advices on how to cope with the extreme food-seeking behavior. Nonetheless, these lifestyle treatments are often insufficient on the long term in both children and adults with genetic obesity, as their hyperphagia remains to be untreated (20).

Additional pharmacotherapy, targeting hyperphagia specifically, is often needed. Next to inducing weight loss and improved mobility, patients often report that decreasing their appetite also improved their quality of life significantly (16, 21, 22). Importantly, the treatment aims for patients with genetic obesity may differ compared to patients with common obesity. For example, weight reduction as well as stabilizing body weight by discontinuing the otherwise progressive weight gain can be considered a treatment goal. Additionally, ameliorating the hyperphagia and, consequently, enhancing their quality of life is considered to be another important treatment goal.

Currently, targeted and nontargeted pharmacotherapies for obesity treatment are available. The nontargeted antiobesity medications (AOM), such as naltrexone-bupropion and glucagon-like peptide-1 (GLP-1) receptor agonists, have been approved for obesity treatment in general, whereas the targeted pharmacotherapeutical agent, setmelanotide, has only been approved for specific genetic obesity disorders. The effects of these nontargeted antiobesity agents in patients with genetic obesity have only been described in case reports, case series, or small cohort studies (21-23). Consequently, the effectiveness in this specific patient group remains unclear. Off-label pharmacotherapeutical agents, such as leptin analogues or central nervous system stimulants, have been described in case series as well for genetic obesity treatment (24-26). Bariatric surgery is another effective but invasive treatment option for obesity, carrying risks of postsurgical complications (27). The long-term effects of bariatric surgery on weight and appetite in genetic obesity remain questionable and therefore should only be considered as a last-resort option, particularly in the era of rapidly evolving, highly effective AOMs for common obesity (28, 29). All in all, this demonstrates that noninvasive and effective appetite-suppressing agents are needed for patients with genetic obesity to induce weight loss, ameliorate hyperphagia, and improve their health and quality of life.

Prader-Willi syndrome (PWS), though a syndromic cause of obesity, is a unique and complex disorder with distinct features that makes comparison to other forms of genetic obesity difficult. This syndrome includes a wide range of symptoms and medical challenges that require specific treatment approaches. Extensive research on weight management in PWS, including the use of approved AOMs, has shown no clear long-term benefits (30, 31). Additionally, several trials are currently investigating new agents such as beloranib, MC4R agonists, and diazoxide choline controlled-release tablets (32). For the previously mentioned reasons, we have chosen to exclude PWS from the results for the remainder of this review.

In this review, we will especially focus on the effects of the currently US Food and Drug Administration (FDA) and European Medicines Agency (EMA) approved AOMs in patients with genetic obesity. Additionally, a summary of off-label drugs used in the past to treat the hyperphagia and obesity in patients with genetic obesity will be provided. Finally, some of the new expected AOMs and their potential role in the future will be described.

## Currently Approved Targeted Antiobesity Agent

Currently, one targeted AOM (ie, setmelanotide) is available that specifically targets the leptin-melanocortin pathway. Studies have shown little effect on weight of setmelanotide in patients with common obesity. However, in patients with specific types of genetic obesity significant effects on weight and satiety were observed. Consequently, setmelanotide is only approved and available for patients with specific genetic obesity disorders (Table 1). All studies reporting the effects of setmelanotide in patients with genetic obesity disorders is summarized in Table 2. Most of the studies investigating setmelanotide are nonrandomized controlled trials.

**Table 1.** Available targeted antiobesity medication

Antiobesity medication	Recommended dose and administration	Approval	For genetic obesity disorders	Approved age categories
Setmelanotide	3.0 mg QD, SC	FDA, EMA	Biallelic POMC, LEPR, PCSK1 deficiency Bardet Biedl syndrome	FDA and EMA: adults, children ≥6 years

Abbreviations: EMA, European Medicines Agency; FDA, U.S. Food and Drug Administration; LEPR, leptin receptor; mg, milligram; PCSK1, proprotein convertase subtilisin and kexin type 1; POMC, Pro-opiomelanocortin; QD, daily; SC, subcutaneous.

**Table 2.** Overview of studies reporting outcomes of setmelanotide treatment in patients with genetic obesity disorders

No.	Reference	Gene defect	Medication	Study characteristics	Side effects	Duration treatment	Summary outcome
1.	Collet et al, 2017 (33)	Heterozygous *MC4R*	Setmelanotide SC, 0.01 mg/kg/24 hours	NRCT, n = 9, aged 22-57 years	56.4% skin discoloration, 33.3% headache, 17.9% skin lesions, 15.4% nausea, 12.8% penile erections, 12.8% infusion site pain, 10.3% diarrhea	28 days	Weight: -3.48 kg (95% CI, -4.99 to -1.96), nonsignificant compared to placebo Vital functions: unchanged Metabolic: waist circumference -5.83 cm (95% CI, -9.70 to -1.96), nonsignificant compared to placebo BMI, body compositions, appetite: others: N.R.
2.	Kuhnen et al, 2016 (34)	Biallelic *POMC*	Setmelanotide SC, individualized dose ranging between 0.5-1.5 mg/day	NRCT, n = 2, aged 21 and 26 years	Dry mouth, injection site reaction, skin hyperpigmentation, darkening hair, mild episodes of fatigue, sadness or emptiness	Patient 1: 42 weeks, patient 2: 12 weeks	Weight: patient 1 -51.0 kg (-32.9%), patient 2 -20.6 kg (-13.4%) BMI: patient 1 -16.4 kg/m^2^, patient 2 −7.2 kg/m^2^ Body composition: patient 1 FM -41.1 kg, LBM -11.2 kg, patient 2 FM -17.9 kg, LBM -2.6 kg Vital functions: patient 1 decrease in BP and HR, unchanged in patient 2 Metabolic: improved lipids / hyperinsulinemia, reduced leptin Appetite: improvement hyperphagia using Likert hunger score Others: REE/LBM (kcal/kg) patient 1 -8.4, patient 2 -3.9
3.	Clémente et al, 2018 (35)	Biallelic *LEPR*	Setmelanotide SC, individualized dose ranging between 1.5-2.5 mg/day	NRCT, n = 3, aged 22, 23, and 14 years	Skin hyperpigmentation, darkening hair	24-61 weeks	Weight: patient 1 -25.1 kg (-19.2%), patient 2 -12.0 kg (-9.8%), patient 3 -7 kg (-5.8%) Vital functions: decreased SBP and HR in patient 1 en 3, others unchanged Metabolic: trend to normalization of hyperinsulinemia Appetite: reduced hyperphagia in every patient BMI, body composition, others: N.R.
4.	Haws et al, 2020 (36)	*BBS*	Setmelanotide SC, individualized dose, maximum of 3.0 mg/day	NRCT, n = 10, mean age 22.5 ± 14.7 years (6 patients 12-19, 4 patients >18)	100% injection site reaction, 80% hyperpigmentation	52 weeks	Weight: -5.5% (90% CI, -9.3% to -1.6%; n = 8) at 3 months, -11.3% (90% CI, -15.5% to -7.0%; n = 8) at 6 months, -16.3% (90% CI, -19.9% to -12.8%; n = 7) at 12 months BMI: 3 months -5.5%; n = 8, 6 months (-11.1%; n = 8) and 12 months -16.2%; n = 7 Body composition: FM 3 months -9.3%, 12 months −24.0% Vital functions: BP and HR unchanged Metabolic: lipids and glucose unchanged Appetite: reduction hunger scores Others: N.R.
5.	Argente et al, 2021 (37)	*SH2B1* and distal 16p11.2 deletion syndrome	Setmelanotide SC, individualized dose, maximum of 3.0 mg/day	NRCT, n = 35, of which 22 with SH2B1 and 13 with distal deletion, age N.R.	71.4% skin hyperpigmentation, 48.6% nausea, 37.1% Headache. AEs led 9 patients (25.7%) to discontinue the study	12 weeks	Weight: adults, 8 responders -7.2% ± 2.1% [SH2B1 variants, n = 5; 16p11.2, n = 3], 14 non-responders -0.8% ± 2.3, overall -3.1% ± 3.9%. Children, 7 responders BMI z-score -0.25 ± 0.06 [SH2B1 variants, n = 5; 16p11.2, n = 2], 6 non-responders 0.03 ± 0.06, overall -0.15 ± 0.13 BMI, body composition, vital functions, metabolic, appetite, others: N.R.
6.	Farooqi et al, 2021 (38)	*SRC1*	Setmelanotide SC, individualized dose, maximum of 3.0 mg/day	NRCT, n = 30, 10 children, 20 adults, mean age 30.6 years (range, 9.0-66.0)	66.7% skin hyperpigmentation, 36.7% nausea, 30.0% injection site bruising, 1 patient withdrew due to AE	12 weeks	Weight: adults, 6 responders = -7.9% ± 2.2%, 14 nonresponders -2.3% ± 1.9, overall -3.9% ± 3.3%. Children, 3 responders BMI *Z*-score -0.48 ± 0.28, 7 nonresponders -0.09 ± 0.03, overall -0.21 ± 0.23 BMI, body composition, vital functions, metabolic, appetite, others: N.R.
7.	Clement et al, 2020 (39)	Biallelic *POMC*, *PCSK1* or *LEPR*	Setmelanotide SC, individualized dose, maximum of 3.0 mg/day	NRCT, n = 10 with POMC or PCSK1 (mean age 18.4 ± 6.2 years), n = 9 LEPR (mean age 23.7 ± 8.4 years)	POMC: injection site reaction, hyperpigmentation, nausea, vomiting, spontaneous penile erections, 1 patient with grade 1 hypereosinophilia	12 months	Weight: POMC -25.6% ± 9.9, LEPR 12.5% ± 8.90 Body composition: POMC FM -38.6% ± 15.4, LBM -10.7% ± 8.2, LEPR FM -15.0% ± 14.6, LBM -7.4% ± 5.1 Vital functions: unchanged Metabolic: POMC unchanged glycemic indices, improved fasting glucose, improved HDL and triglycerides. LEPR unchanged glycemic indices, improved HDL and LDL Appetite: hunger score POMC -27.1% ± 28.1, LEPR-43.7% ± 23.7 Others: -
8.	Clement et al, 2022 (40)	Biallelic *POMC* or *LEPR*	Setmelanotide SC, individualized dose, maximum of 3.0 mg/day	NRCT, n = 24, age N.R.	No new safety issues	36 months	BMI: total group 12 months -24.8% ± 8.2 (n = 24), 24 months -21.0% ± 13.0 (n = 23), 36 months -24.0% ± 17.9 (n = 15). Adults, 12 months -25.1% ± 7.7 (n = 11), 24 months -22.9% ± 12.5 (n = 11), 36 months -24.4% ± 13.2 (n = 8). Children, BMI *Z* score 12 months -1.31 ± 0.66 (n = 13), 24 months -1.10 ± 0.79 (n = 11), 36 months -1.01 ± 1.22 (n = 4) Weight, body composition, vital functions, metabolic, appetite, others: N.R.
9.	Haqq et al, 2022 (41)	*BBS* or *ALMS1*	Setmelanotide SC, individualized dose, maximum of 3.0 mg/day	NRCT, n = 32 BBS and n = 6 Alström, mean age 19.9 ± 10.2 years	61% skin hyperpigmentation, 48% injection site erythema, 2 patients SAEs (blindness, anaphylactic reaction, and suicidal ideation)	52 weeks	Weight: BBS + ALMS -5.9 kg ± 9.3 (-5.2% ± 7.9), only BBS -7.4 kg ± 8.2 (-6.5% ± 7.0) BMI: only BBS BMI -4.2 kg/m^2^ ± 3.3, BMI *Z*-score -0.8 ± 0.5, P95BMI% -17.3% ± 7.7 Body composition: BBS + ALMS FM -4.6 kg ± 12.0 (-8.8% ± 26.1), FFM -0.7 kg ± 4.0 (-1.0% ± 7.0), only BBS FM -5.6 kg ± 12.0 (-11.3% ± 26.3), FFM -1.2 kg ± 3.9 (-2.0% ± 6.5) Metabolic: improved lipids in both BBS and ALMS Appetite: reduction maximal hunger score in patients aged ≥ 12 years -2.3 (-30.9%) in BBS + ALMS, -2.1 (-30.5%) in only BBS Vital functions, others: N.R.
10.	Wabitsch et al, 2022 (15)	Biallelic *POMC* or *LEPR*	Setmelanotide SC, dose N.R.	NRCT, n = 3 with POMC, n = 2 with LEPR, mean age 23.8 years (range, 15.0-33.0)	N.R.	3-4 years	Weight: mean decrease of -47.4 kg (range, 25.0-97.0) Appetite: reduction hunger, a decrease in the intensity as well as the frequency and duration of their hunger, decreased food intake, increased satiation Others: improved QoL, improved physical activity BMI, body composition, vital functions, metabolic: N.R.
11.	Kuhnen et al, 2022 (42)	Biallelic *POMC* or *LEPR*	Setmelanotide SC, individualized dose, maximum of 3.0 mg/day	NRCT, n = 16, aged 11-37 years	N.R.	12 months	Weight: adults -21.2% ± 11.3, children aged 8-12 years -11.2% ± 12.5, children aged 13-17 years -29.8% ± 4.2 Appetite: hunger score adults -43.5% ± 28.7, children aged 13-17 years -24.3% ± 26.2 Others: 5 out of 6 adults clinically meaningful improvement in IWQOL-Lite (+24.2 ± 12.1), 3 out of 5 children clinically meaningful improvement in PedsQL, PHQ-9 in participants aged > 12 years: unchanged BMI, body composition, vital functions, metabolic: N.R.
12.	Kühnen and Clément 2022 (43)	Biallelic *POMC*	Setmelanotide SC, individualized dose, maximum of 3.0 mg/day	NRCT, n = 2, aged 21 and 26 years	Persistent skin hyperpigmentation, darkening hair	7.2 and 6.8 years	Weight: patient 1 -55.6 kg (-35.8%), patient 2 -72.6 kg (-47.5%) BMI: patient 1 -17/7 kg/m^2^, patient 2 -25.3 kg/m^2^ Body composition: patient 1 FM -39.3 kg, LBM -12.4 kg, patient 2 FM -66.4 kg, LBM -6.2 kg Vital functions: unchanged BP and HR Metabolic: normalized hyperinsulinemia, improved glycemic indices, improved lipids Appetite: decrease hunger scores Others: improvement QoL due to normalization of hunger and body weight, REE/kg patient 1 from 15.54 > 19.3, patient 2 from 13.9 > 20.1
13.	Argente al a., 2022 (44)	*SH2B1* and distal 16p11.2 deletion syndrome	Setmelanotide SC, individualized dose, maximum of 3.0 mg/day	NRCT, n = 35, of which 22 with SH2B1 and 13 with distal deletion, age N.R.	No new safety concerns	12 months	Weight: adults -4.4% ± 5.0 (n = 9), -6.8% ± 5.0 (n = 7), and -7.7% ± 10.0 (n = 8) after 6, 9, and 12 months, resp. BMI: total group -3.4% ± 8.1 (n = 19), -5.9% ± 10.0 (n = 15), and -9.7% ± 8.0 (n = 14) after 6, 9, and 12 months of treatment, children BMI *Z*-score -0.55 ± 0.17 (n = 6) after 12 months Others: only patients included who experienced clinical benefit in first year of treatment Body composition, vital functions, metabolic, appetite: N.R.
14.	Martos-Moreno et al, 2022 (45)	*SRC1*	Setmelanotide SC, individualized dose, maximum of 3.0 mg/day	NRCT, n = 17, age N.R.	No new safety concerns	12 months	Weight: adults -6.7% ± 6.1 (n = 11), -9.9% ± 7.4 (n = 8), and -11.0% ± 8.6 (n = 7) after 6, 9, and 12 months, resp. BMI: total group -5.7% ± 5.6 (n = 16), -7.8% ± 5.8 (n = 11), and -10.1% ± 9.4 (n = 8) after 6, 9, and 12 months resp. Children BMI *Z*-score -0.35 ± 0.35 (n = 7), -0.42 ± 0.23 (n = 5), -0.67 ± 0.57 (n = 3) after 6.9 and 12 months resp. Others: only patients included who experienced clinical benefit in first year of treatment Body composition, vital functions, metabolic, appetite: N.R.
15.	Dubern et al, 2023 (46)	Biallelic *POMC*	Setmelanotide SC, 0.5 mg/day	CR, n = 1, aged 5 years	Persistent skin hyperpigmentation, transient erections during first month of treatment	12 months	Weight: -30 kg (-36%) BMI: -15.3 kg/m^2^ (40.7 > 25.4), BMI SD −12 (18 > 6) Body composition, vital functions: N.R. Metabolic: 30% improvement of lipid values, liver enzymes, and fasting insulin Appetite: decrease hyperphagia (-48% decrease in Dykens score, -18% decrease in CEBQ) Others: decreased emotional impact (-60%) on parents quality of life
16.	Forsythe et al, 2023 (47)	*BBS*	Setmelanotide SC, individualized dose, maximum of 3.0 mg/day	NRCT, n = 20, mean age 22.0 ± 10.9 years	N.R.	52 weeks	Weight, body composition, vital functions, metabolic: N.R. BMI: adults -9.4% ± 6.7, children BMI *Z*-score -0.7 ± 0.5 Appetite: 3 children -52.7% ± 23.2 in hunger score, 7 adults -39.6% ± 21.5 in hunger score Others: 9 children PedsQL +11.2 ± 14.3, 11 adults IWQOL-Lite +12.0 10.3
17.	Argente et al, 2023 (48)	*BBS*	Setmelanotide SC, individualized dose, maximum of 3.0 mg/day	NRCT, n = 30, age N.R.	No new safety concerns	24 months	Weight: adults -8.6% ± 10.3 (n = 15), -14.9% ± 10.4 (n = 6), and -7.7% ± 10.0 (n = 8) after 18 and 24 months, resp. BMI: total group -9.5% ± 10.5% (n = 30), -14.3% ± 11.6 (n = 19), and -9.7% ± 8.0 (n = 14) after 6, 9, and 12 months of treatment, children BMI *Z*-score -0.83 ± 0.50 (n = 13) and -0.72 ± 0.54 (n = 12) after 18 and 24 months, resp. Others: only patients included who experienced clinical benefit in first year of treatment Body composition, vital functions, metabolic, appetite: N.R.
18.	Clément et al, 2023 (49)	Biallelic *POMC* or *LEPR*	Setmelanotide SC, individualized dose, maximum of 3.0 mg/day	NRCT, n = 24, age N.R.	No new safety concerns	24 months	Body composition: FM -37.2% ± 13.0 (n = 22) and -32.4% ± 16.7 (n = 17) at 12 and 24 months, LBM -7.8% ± 10.8 and -6.5% ± 16.2 at 12 and 24 months Others: only patients included who experienced clinical benefit (adult ≥10% weight loss, children ≥0.3 BMI *Z* score reduction) in first year of treatment Weight, BMI, vital functions, metabolic, appetite: N.R.
19.	Haqq et al, 2023 (50)	*BBS*	Setmelanotide SC, individualized dose, maximum of 3.0 mg/day	NRCT, n = 22, age ranges 10-44 years	No new safety concerns	24 months	Metabolic: total group, responders MetS-*Z*-BMI -0.54 ± 0.56 (n = 16) and nonresponders MetS-*Z*-BMI +0.17 ± 0.50 (n = 6) after 52 weeks of treatment. Only children, responders MetS-*Z*-BMI -0.38 ± 0.53 (n = 9) and nonresponders MetS-*Z*-BMI +0.42 ± 0.38 (n = 4) after 52 weeks of treatment Weight, BMI, vital functions, body composition, appetite, others: N.R.
20.	Wabitsch et al, 2023 (51)	Biallelic *POMC* or *LEPR*	Setmelanotide SC, individualized dose, maximum of 3.0 mg/day	NRCT, n = 9, age N.R	N.R.	52 weeks	Metabolic: Responders (≥-0.3 BMI *Z*-score): MetS-*Z*-BMI -1.18 ± 0.72 (n = 8) Nonresponder: MetS-*Z*-BMI −0.34 (n = 1 with -0.1 BMI *Z*-score) Weight, BMI, vital functions, body composition, appetite, others: N.R.
21.	Lazareva et al, 2024 (52)	SMS	Setmelanotide SC, individualized dose, maximum of 3.0 mg/day	NRCT, n = 12, age ranges 11-39 years	No new safety concerns	4 months	Weight: -0.31 kg (95% CI, -1.81 to 1.19, NS), corresponding with -0.28% BMI: -0.27 kg/m^2^ (95% CI, -0.98 to 0.45, NS), corresponding with -0.63% Body composition: FM -0.88 kg (95% CI, -3.65 to 1.88,NS), lean mass 1.64 kg (95% CI, -0.69 to 3.96, NS) Vital functions: non-significant decreases in SBP/DBP Metabolic: significant decrease in total cholesterol and HDL-cholesterol, trend for lower LDL-cholesterol, nonsignificant decrease triglycerides, glucose, HbA1c, and leptin Appetite: significant reduction in the median overall hunger score Others: N.R.

Abbreviations: AE, adverse event; BBS, Bardet-Biedl syndrome; BMI, body mass index; BP, blood pressure; CEBQ, Children's Eating Behavior Questionnaire; CR, case report; CS, case series; EE, energy expenditure; FFM, fat-free mass; FM, fat mass; HDL, high-density lipoprotein; HR, heart rate; IWQOL-lite, Impact of Weight on Quality of Life-Lite; LBM, lean body mass; LDL, low-density lipoprotein; LEPR, leptin receptor; MC4R, melanocortin-4 receptor; MetS-*Z*-BMI, metabolic syndrome severity *Z*-score with BMI as measure for adiposity; N.R, not reported; NRCT, nonrandomized controlled trial; PCSK1, Proprotein convertase subtilisin/kexin type 1; PedsQL, Pediatric Quality of Life Inventory; PHQ-9, Patient Health Questionnaire-9; POMC, pro-opiomelanocortin; QoL, quality of life; REE, resting energy expenditure; SBP, systolic blood pressure; SH2B1, SH2B adaptor protein 1; SMS, Smith Magenis Syndrome; SRC1, steroid receptor coactivator-1.

### Setmelanotide

#### Mechanism of action

The MC4R, embedded within the leptin-melanocortin pathway, plays a pivotal role in the regulation of appetite and body weight (Fig. 1). The first generations of MC4R agonists were, however, either ineffective or led to significant adverse effects, such as increased blood pressure (11). Setmelanotide was the first MC4R ligand with weight-reducing potential but without severe adverse effects. It is a potent and selective MC4R agonist, which upon binding restores MC4R function. By specifically targeting the MC4R, the genetic defects located upstream are bypassed (11).

#### Effects in common obesity

Although studies in rodents with diet-induced obesity treated with MC4R agonists have shown promising results, these results have not been observed in humans with obesity (53). A randomized controlled trial (RCT), using MK-0493, which is also a potent MC4R agonist, concluded that treatment with this MC4R agonist did not lead to weight loss (-1.2 kg; 95% CI, -2.6 to 0.2) after 18 weeks of treatment (54). Interestingly, an RCT investigating the impact of 72 hours of continuous subcutaneous infusion of setmelanotide on resting energy expenditure, showed a +6.4% (95% CI, 0.68-13.0) increase in resting energy expenditure (55). To our knowledge, no other studies have been published using setmelanotide as obesity treatment in patients with common obesity.

#### Effects in genetic obesity

Two young adults with POMC deficiency were the first to be treated with setmelanotide resulting in impressive effects on body weight (-32.9% after 42 weeks and -13.4% after 12 weeks), body composition, cardiometabolic parameters, and, perhaps most importantly, hyperphagia (34). A case series, including three adults with LEPR deficiency, followed, revealing a weight-reducing effect ranging between -5.8% to -19.2%, improved cardiometabolic parameters and hyperphagia after 24 to 61 weeks of treatment (35). A phase 3 nonrandomized trial followed in which slightly larger numbers of patients, including both children aged ≥6 years and adults, with POMC, PCSK1, and LEPR deficiency were treated, resulting in a mean decrease of -25.6% ± 9.9 in patients with POMC or PCSK1 deficiency and -12.5% ± 8.9 in patients with LEPR deficiency (39). A decrease of -1.6 ± 0.9 and -0.5 ± 0.4 in BMI *Z*-score was observed after 12 months of treatment in the subgroup of children with POMC (n = 6) and LEPR deficiency (n = 3), respectively (39). Moreover, reduced metabolic syndrome severity score, implying a reduced risk of metabolic syndrome, cardiovascular disease, and type 2 diabetes (T2D), improved appetite regulation and quality of life were reported during setmelanotide treatment in these patients (15, 42, 51). Currently, studies are ongoing to assess if setmelanotide might also be safe and effective to treat children with genetic obesity younger than 6 years (NCT04966741). A case report in a 5-year-old child with POMC deficiency already demonstrated a -36% weight loss, improved hyperphagia and metabolic parameters, and no serious side effects during 12 months of setmelanotide treatment (46). As the first study was published in 2016, some long-term data are available. A follow-up study of the 2 first described adults with POMC deficiency demonstrated long-term sustained weight-reducing effects of setmelanotide: -35.8% and -47.5% after, respectively, 7.2 and 6.8 years of treatment (34, 43). Long-term efficacy was also described in a conference paper, reporting a weight decrease of -24.0% ± 17.9 after 36 months of treatment in 15 patients with biallelic POMC or LEPR deficiency (40). Body composition analyses showed a fat mass decrease of -32.4% ± 16.7 (n = 17) together with a less favorable lean body mass decrease of -6.5% ± 16.2 in 17 patients with biallelic POMC or LEPR deficiency after 24 months of treatment (49). Also in patients with BBS and AS the weight-reducing potential of setmelanotide was observed in phase 2 and 3 nonrandomized trials. The phase 2 trial, with a total of 10 patients (6 children and 4 adults) with BBS, showed a mean weight reduction of -11.3% and -16.3% after resp. 6 and 12 months of treatment (36). Additionally, average hunger score (-63.9% ± 16.5), maximal hunger score (-70.2% ± 17.2), fat mass (-24.0%), and waist circumference (-17.0%) decreased significantly after 12 months of treatment (36). The phase 3 trial, including a 14-week randomized placebo-controlled period, showed similar results in a larger group of patients including children aged ≥6 years and adults with BBS: a mean weight decrease of -7.6% ± 7.1 in the adults, a -17.3% ± 7.7 decrease in BMI percent of the 95th percentile (BMI%P95) in children, decreases in maximal hunger score (-30.5% ± 26.5 in patients aged ≥12 years), and improvements in health-related quality of life measures after 52 weeks of treatment (41, 47). For patients with AS, the results were inconclusive (41). Sustained weight-reducing effects and a reduced metabolic syndrome severity score were observed for children and adults with BBS after 24 months of follow-up (48, 50). The weight-reducing potential of setmelanotide was not observed in an RCT including 9 patients with heterozygous MC4R deficiency and an open-label study including 12 patients with Smith Magenis syndrome (33, 52).

One of the most outstanding and frequently reported side effects is hyperpigmentation of skin, lips, and naevi, which is related to cross stimulation of the MC1R (56). Until now, malignant lesions have not been reported; however, studies with longer follow-up are needed to determine whether this risk is altered (43). Other most commonly reported side effects are injection site reactions, such as pain, erythema and pruritus, nausea, vomiting, headache, and spontaneous penile erections (33, 36, 39, 41, 44). No treatment-related effects on suicidality or depression have been observed in 13 patients with POMC or LEPR deficiency during setmelanotide treatment (57).

In conference articles, varying effects on weight, identifying responders and nonresponders, during setmelanotide treatment in patients with defective SH2B1 or SRC1 has been reported (37, 38). In the patients who initially responded to setmelanotide treatment, a sustained weight-reducing effect was observed after 12 months of treatment (45, 58). Currently, multiple phase 2 and 3 trials are being performed investigating the impact of setmelanotide treatment in patients with obesity from defects in various genes located in the leptin-melanocortin pathway, with the final aim to extend the indication of setmelanotide treatment to a larger group of patients with genetic obesity (NCT05093634, NCT04963231, and NCT04966741).

## Currently Approved Nontargeted Antiobesity Medications

A complete overview of the approved nontargeted AOMs worldwide is shown in Table 3. Orlistat, short-term use phentermine (≤12 weeks), phentermine-topiramate, naltrexone-bupropion, high-dosed liraglutide and semaglutide, both GLP-1 analogues, and tirzepatide, a dual GLP-1/glucose-dependent insulinotropic polypeptide (GIP) receptor agonist, have been approved by the FDA for adult obesity treatment. The EMA has approved orlistat, naltrexone-bupropion, liraglutide, semaglutide, and tirzepatide for adult obesity treatment. For children aged 12 years and older with obesity, orlistat, phentermine-topiramate, liraglutide, and semaglutide has been approved by the FDA for obesity treatment, whereas the EMA only approved liraglutide and semaglutide for this pediatric patient group. The FDA has also approved short-term use of phentermine for children aged ≥16 years. All studies reporting the effects of these nontargeted AOMs in patients with genetic obesity disorders are summarized in Table 4. All studies reporting on the effects of the approved nontargeted AOMs in patients with genetic obesity are observational in nature, including case reports, case series, and small cohort studies.

**Table 3.** Available regular nontargeted antiobesity medications

Antiobesity medication	Recommended dose and administration	Approval	Approved age categories
Lipase inhibitors	120 mg TID, oral	FDA, EMA	FDA: adults, children ≥12 years EMA: adults
Phentermine	37.5 mg QD, oral, max. ≤ 12 weeks	FDA	FDA: adults, children ≥16 years
Phentermine-topiramate	15 mg / 92 mg QD, oral	FDA	FDA: adults, children ≥12 years
Naltrexone-bupropion	16 mg / 180 mg BID, oral	FDA, EMA	FDA and EMA: adults
Liraglutide	3.0 mg QD, SC	FDA, EMA	FDA and EMA: adults, children ≥12 years
Semaglutide	2.4 mg QW, SC	FDA, EMA	FDA and EMA: adults, children ≥12 years
Tirzepatide	15.0 mg QW, SC	FDA, EMA	FDA and EMA: adults

Abbreviations: BID, 2 times per day; EMA, European Medicines Agency; FDA, U.S. Food and Drug Administration; QD, daily; QW, weekly; TID, 3 times per day.

**Table 4.** Overview of studies reporting outcomes of nontargeted agents in patients with genetic obesity disorders

No.	Reference	Gene defect	Medication	Study characteristics	Side effects	Duration treatment	Summary outcomes
*Approved antiobesity medications*							
1.	Iepsen et al, 2018 (23)	Heterozygous *MC4R*	Liraglutide SC, 3.0 mg/day	NRCT, n = 14, mean age 32.8 ± 13.5 years	Week 1-4: nausea in 57.1%, after 16 weeks nausea in 14.3%	16 weeks	Weight loss: -6.8 ± 1.8 kg (-5.7% ± 1.4%) BMI: −2.0 ± 0.5 kg/m^2^ Body composition: FM -5.0 kg ± 1.5 (-2.5% ± 0.8%), FFM -0.9 kg ± 0.4 Vital functions: DBP -1.5 ± 2.0, HR +8.6 ± 2.4 Metabolic: decreased fasting glucose, improved lipids Appetite: reduced appetite
2.	Iepsen et al, 2020 (59)	Homozygous *MC4R*	Liraglutide SC, 3.0 mg/day	CR, n = 1, aged 17.9 years	Nausea, stomach pain during first 4 weeks of treatment	16 weeks	Weight loss: -9.7 kg BMI -3.6 kg/m^2^ Body composition: FM -4.3 kg (=0.3%), FFM -5.6 kg Metabolic: lowering of SBP, improved glycemic control and lipid levels Appetite: less hungry and more satiated during week 1-8, afterwards still present but less
3.	Klee et al, 2020 (60)	Homozygous *MC4R*	Liraglutide SC, 3.0 mg/day	CR, *n* = 1, aged 16 years	None	2 months	Weight: -4 kg BMI: -4.4 kg/m^2^ Appetite: decreased Metabolic: HbA1c -0.4%, HOMA-IR -2.16 Vital functions, body composition, others: N.R.
4.	Welling et al, 2021 (21)	Heterozygous *MC4R*, 16p11.2 deletion	Exenatide SC, 20 μg/day, liraglutide SC, 3.0 mg/day	CS, n = 4, adults (age N.R.)	Two patients experienced mild side effects for a brief period	43 weeks—12 years	Weight loss: ranging between -10.5 kg to -31.2 kg (-6.1% to -27.6%) BMI: ranging between -2.1 to -10.6 kg/m^2^ Metabolic: improved dyslipidemia in n = 1, improved glycemic control in n = 1 Appetite: improved satiety in n = 3 Vital functions, body composition, and others: N.R.
5.	Çamtosun et al, 2021 (61)	Heterozygous *MC4R*	Liraglutide SC, 1.8 mg/day	CR, n = 1, aged 17.9 years	Nausea, bloating, belching, intermittent abdominal pain, and gas-related pain	32 weeks	Weight loss: -27.8 kg (-19.2%) BMI -10.15 kg/m^2^ Appetite: reduced appetite Others: full weight regain after cessation of treatment Body composition, metabolic: N.R.
6.	Ganawa et al, 2022 (62)	Homozygous *BBS-10*	Liraglutide SC 1.8 mg/day, semaglutide SC 1.0 mg/week	CR, n = 1, aged 28 years	N.R.	40 months	Liraglutide for 5 months: -9.5 kg weight (-10.4%), BMI -5.5 kg/m^2^ Semaglutide for 19 months: -17.5 kg weight (-21.5%), BMI -9.2 kg/m^2^ Metabolic: improved glycemic control Others: in next 15 months increase BMI of +2 kg/m^2^
7.	Zaitoon et al, 2023 (63)	Homozygous *MC4R*	Liraglutide SC, 3.0 mg/day, metformin, 850 mg/day	CR, n = 1, aged 9 years	Nausea and abdominal pain during dose titration period	1 year	Weight: stabilization of weight BMI: -0.9 kg/m^2^, -14% of BMI%P95 Vital functions: N.R. Body composition: FM -1.3% Metabolic: improved insulin resistance, liver enzymes, lipid profile Appetite: less preoccupied with food, improved appetite control Others: improved concentration at school, greater participation in after-school activities
8.	Ali et al, 2023 (64)	*ALMS*	Semaglutide 14 or 21 mg mg/day or 1.0 mg/week, exenatide 20 mcg/day	Cohort, n = 30, mean age 31 ± 11 years	Nausea, abdominal discomfort and diarrhea during the dose titration period in 9/21 patients	6 months	Weight: mean 5.4 kg ± 1.7 (95% CI 3.6-7), mean -6.0% ± SD N.R. BMI: mean -2.0 kg/m^2^ ± SD N.R. Vital functions: decreased SBP, DBP unchanged Metabolic: improved HbA1c, lipids and ALT Appetite: improved satiety VAS score Body composition, others: N.R.
9.	Gokul et al, 2023 (65)	Heterozygous *MC4R*	Semaglutide 1.0 mg/week	CR, n = 1, aged 13 years	N.R.	6 months	Weight: -10.7 kg (-5.7%) and -18.4 kg (-9.8%) after 3 and 6 months BMI: -4.3 kg/m^2^ Body composition: FM -10.8% Vital functions, metabolic, appetite, others: N.R.
10.	Schirmer et al, 2023 (66)	Homozygous *MC4R* (n = 2), heterozygous *POMC* (n = 1), proximal 16p11.2 deletion (n = 1), heterozygous *NTRK2*(n = 1)	Liraglutide SC, 3.0 mg/day	CS, n = 5, age N.R.	Nausea, vomiting, stomach pain and obstipation in n = 5 during dose escalation phase	9 months	Weight: -8.2% ± 4.3 after 6 months, -9.5% ± 7.3 after 9 months BMI: -0.29 ± 0.20 BMI-SDS after 6 months, -0.34 ± 0.31 BMI-SDS after 9 months Appetite: significant reduction of subjectively reported hunger feelings Vital functions, body composition, metabolic, others: N.R.
11.	Welling et al, 2024 (19)	Heterozygous *MC4R* (n = 8), biallelic *POMC* (n = 1), 16p11.2 deletion (n = 7), *BBS* (n = 3), *GNB1* (n = 2), *PHIP* (n = 1), *TRIP12* (n = 1)	Liraglutide 3.0 mg/day or naltrexone-bupropion	Cohort, n = 23, median age total group 23.0 (IQR 20.0–31.0)	N.R.	16 weeks	Weight: -4.7% (IQR -6.0 to -1.5) for liraglutide treated group and -5.2% ± 5.8 for naltrexone-bupropion Body composition: -6.6 kg (IQR -8.3 to -5.1) in FM for naltrexone-bupropion treated group Metabolic: improved fasting glucose and HbA1c for liraglutide treated group, resolution of obesity-related comorbidities in both liraglutide and naltrexone-bupropion treated group Appetite: improved self-reported appetite in 83.3% of liraglutide treated group and 90.9% of naltrexone-bupropion treated group Others: improved domains of QoL and eating behavior for liraglutide treated group BMI, vital functions: N.R.
12.	Welling et al, 2023 (16)	Heterozygous *MC4R*	Naltrexone-bupropion, 16/180 mg/day	CR, n = 1, aged 31 years	Severe nausea at dosage higher than 16/180 mg	17 months	Weight: -49.8 kg (-26.7%) BMI: -14.8 kg/m^2^ Body composition: FM -39.9 kg (-38.3%) Vital functions: unchanged Metabolic: normalized fasting insulin, decrease leptin from >100 μg/L to 45.7 μg/L Appetite: diminished hyperphagia Others: improved physical fitness, quality of life, and emotional well-being (all self-reported)
13.	Salazar-Valencia et al, 2022 (67)	Heterozygous *MC4R*	Phentermine, 30 mg/day	CS, n = 6, adults (age N.R.)	83.3% nausea / dry mouth, 66.7% dizziness, anxiety/headache 50%, GI symptoms	6 months	Weight loss: -12.7 kg ± 2.3, 15.5% ± 2.9 Body composition: FM -4.6% ± 2.2, muscle mass 1.2 ± 0.9 Vital functions: SBP +2.0 ± 2.0, DBP +2.0 ± 3.7 Metabolic: reduced glycemic indices, reduced leptin -13.1 ± 7.5 BMI, appetite, others: N.R.
*Off-label pharmacological agents*							
14.	Farooqi et al, 1999 (68)	Biallelic *LEP*	Recombinant human leptin SC, 0.028 mg/kg LBM/day	CR, n = 1, aged 9 years	None	12 months	Weight: -16.4 kg (-17.4%) BMI: -10.1 kg/m^2^ (-21.0%) Body composition: -15.6 kg (-27.9%) fat mass, body fat -7.5%, -0.82 kg lean mass Vital functions: unchanged Metabolic: improved fasting insulin and non-esterified fatty acids Appetite: reduced food intake (-42%), amelioration hyperphagia Others: -340 kcal/day (-18%) REE, -70 kcal/day (-2.4%) TEE, increased physical-activity level
15.	Farooqi et al, 2002 (69)	Biallelic *LEP*	Recombinant human leptin SC, individualized dose	CS, n = 3, aged 3, 4, and 9 years	N.R.	48, 36 and 6 months	Weight: patient 1 -17.5 kg (-18.5%), patient 2 -4.8 kg (-11.7%), patient 3 -1.3 kg (-3.4%) BMI: patient 1 -15.9 kg/m^2^ (-33.0%), patient 2 -14.4 kg/m^2^ (-40.2%), patient 3 -4.8 kg/m^2^ (-12.4%) Body composition: FM patient 1 -18.9 kg (-10.6%), patient 2 -10.7 kg (-20.8%), patient 3 -2.2 kg (-4.1%), FFM patient 1 + 0.7 kg, patient 2 + 5.0 kg, patient 3 + 0.1 kg Vital functions: N.R. Metabolic: improved hyperinsulinemia, and improved lipids Appetite: reduced food intake (ranges 45-84%), amelioration hyperphagia Others: increased thyroid levels, improved circulating CD4+ T cells and impaired T-cell proliferation and cytokine release, REE/ LBM patient 1 -0.03 MJ/kg/day, patient 2 -0.03 MJ/kg/day
16.	Gibson et al, 2004 (70)	Biallelic *LEP*	Recombinant human leptin SC, individualized dose	CR, n = 1, aged 5 years	N.R.	4 years	Weight: -16 kg (-24.8%) BMI: -19.2 kg/m^2^ (-44.2%) Body composition: FM -15.9 kg (-15.3%), FFM -2.4 kg Metabolic: normalized triglycerides and hyperinsulinemia Others: normalization of thyroid biochemistry Vital functions, appetite: N.R.
17.	Licinio et al, 2004 (71)	Biallelic *LEP*	Recombinant human leptin SC, 0.01-0.04 mg/kg/day	CS, n = 3, aged 27, 35, and 40 years	Mild skin reaction for 1 week in 1 patient	18 months	Weight: patient 1 -69.8 kg (-52.1%), patient 2 -48.0 kg (−44.3%), and patient 3 -58.1 kg (-43.9%) BMI: decrease BMI from 51.2 ± 2.5 kg/m^2^ to 26.9 ± 2.1 Body composition: FM patient 1 -52.1 (-88.9%), patient 2 -37.6 kg (-72.3%), patient 3 -39.1 kg (-60.2%), FFM patient 1 -17.7 (-23.4%), patient 2 -10.4 kg (-18.4%), patient 3 -19.0 kg (-28.1%), body fat% patient 1 -33.7%, patient 2 -24.1%, patient 3 -14.8% Vital functions: N.R. Metabolic: patient 1 resolution of hypogonadism, patient 3 resolution of type 2 diabetes without additional antidiabetes pharmacotherapy Appetite: -49% food intake Other: increased physical activity
18.	Paz-Filho et al, 2008 (72)	Biallelic *LEP*	Recombinant human leptin SC, individualized dose	CS, n = 3, aged 32, 42, and 46 years	N.R.	6 years	BMI: patient 1 -28.4 kg/m^2^ (-55.3%), patient 2 -20.3 kg/m^2^ (-43.5%), patient 3 -23.1 kg/m^2^ (-41.7%) Metabolic: improved DM2, improved insulin resistance Appetite: reduced hyperphagia Weight loss, body composition, vital functions, others: N.R.
19.	Galgani et al, 2010 (73)	Biallelic *LEP*	Recombinant human leptin SC, 0.02-0.04 mg/kg/day	CS, n = 3, aged 26, 34, and 39 years	N.R.	19 weeks	Weight: patient 1 -15.8 kg (-11.9%), patient 2 -14.5 kg (-13.9%), patient 3-16.3 kg (-12.3%) Body composition: FM patient 1 -10.3 kg, patient 2 -11.8 kg. patient 3 -13.2 kg, LBM patient 1 -5.5 kg, patient 2 -2.7 kg, patient 3 -3.1 kg Others: 24-hr EE unchanged (in contrast with controls losing weight), increased 24-h fat oxidation BMI, vital functions, metabolic, appetite: N.R.
20.	von Schnurbein et al, 2013 (74)	Biallelic *LEP*	Recombinant human leptin SC, 0.024 mg/kg BW/day	CR, n = 1, aged 14.8 years	N.R.	62 weeks	Weight: -22.9 kg (-22.1%) BMI: -8.5 kg/m^2^ (-24.0%) Body composition: FM -12.3% Vital functions: Metabolic: hepatic lipid content -40.3% (-81.1%), improved lipids, improved liver enzymes, improved glycemic indices Appetite: -21.3% reduction in food intake Others: N.R.
21.	Wabitsch et al, 2015 (75)	Biallelic *LEP*	Recombinant human leptin SC, 0.03 mg/kg LBM/day	CR, n = 1, aged 37 months	N.R.	18 weeks	Weight: -8.3 kg (-19.2%) BMI: -10.4 kg/m^2^ (-23.3%), BMI SDS -0.9 SD (-15.3%) Body composition: FM after 9 weeks -2.6 kg Metabolic: improved glycemic indices, improved lipids, improved liver enzymes, improved leptin Appetite: normalized eating behavior Vital functions, others: N.R.
22.	Roth et al, 2019 (76)	Biallelic *LEP*	Recombinant human leptin SC, 0.023 mg/kg LBM/day	CR, n = 1, aged 14.7 years	N.R.	46 weeks	Weight: -21.4 kg (-20.7%) BMI: -8.5 kg/m^2^ (-24.0%) Body composition: FM -10.8 kg (-21.8%) Metabolic: increase insulin and PYY secretion, reduced ghrelin secretion Appetite: decreased food intake (-21.1% kcal) Vital functions, others: N.R.
23.	Beghini et al, 2019 (77)	Biallelic *LEP*	Recombinant human leptin SC, dose N.R.	CS, n = 9, mean age 8.2 ± 5.4 years	N.R.	12 months	BMI: decrease SDS from +4.1 ± 1.4 to 1.40 ± 0.81 Weight, body composition, vital functions, metabolic, appetite, others: N.R.
24.	Zorn et al, 2022 (78)	Biallelic *LEP*	Recombinant human leptin SC, 0.03 mg/kg LBM, later up dosed	CR, n = 1, aged 9–16 years	N.R.	7 years	BMI: -7.9 kg/m^2^ (-1.15 BMI SD) Metabolic: improvement of most metabolic complications Appetite: improved hyperphagia Weight, body composition, vital functions, others: N.R.
25.	Von Schnurbein et al, 2022 (79)	Biallelic *LEP*	Recombinant human leptin SC, dose N.R.	CS, n = 6, age N.R.	N.R.	4 months	Others: improved indices of physical activity and indices of psychological wellbeing on short and long-term Weight, BMI, body composition, vital functions, metabolic, appetite: N.R.
26.	Torchen et al, 2022 (80)	Biallelic *LEP*	Recombinant human leptin SC, dose 5 mg/day	CS, n = 2, age 18 (A) and 20 (B) years	No serious side effects	18 months	Weight: patient A -45.3 kg (-32.8%), patient B -39.1 kg (-33.1%) BMI, vital functions, appetite: N.R. Body composition: FM (A: -13%, B: -18%) Appetite: improved perceived hunger and dietary disinhibition Metabolic: patient A -3.9% HbA1c (normalized), HOMA2-IR (A: -83%, B: -80%), ALT (A: -90%, B: -91%), triglycerides (A: -47%, B: -26%), hsCRP (A: -80%, B: -84%), FGF-21 (A: -81%, B: -67%), adiponectin (A: + 42%, B: + 37%), and improved hepatic steatosis and BMD in both patients Others: improved mood, self-esteem, physical function, energy, and quality of life, resumed spontaneous menses in both patients
27.	Funcke et al, 2023 (81)	Biallelic *LEP*	Recombinant human leptin SC, max. dose 0.7 (A) and 0.15 (B) mg/kg LBM, later resp. 0.15 and 0.08 mg/kg LBM	CS, n = 2, aged 14 (A) and 2 (B) years	No severe adverse events	39.1 (A) and 41.4 (B) months	Weight: patient A -85.8 kg, patient B -3.1 kg BMI: patient A -27.4 kg/m^2^ and BMI-SDS -4.36, patient B -14.0 kg/m^2^ and BMI-SDS -7.61 Body composition: patient A FM -26.2% after 1 year Vital functions: Metabolic: decreased fasting insulin, HbA1c, and liver enzymes in patient A, decreased ALT in patient B Appetite: hunger score patient A -2.05 Others: increasing anti-metreleptin antibodies in both patients
28.	Hilado and Randhawa, 2018 (82)	Homozygous *POMC*	Metformin, 300-400 mg/day	CR, n = 1, aged 4 years	N.R.	36 months	BMI: decrease from 34.9 to 32.9 kg/m^2^ Weight loss, body composition, vital functions, metabolic, appetite, others: N.R.
29.	Albayrak et al, 2011 (24)	Heterozygous *MC4R*	Methylphenidate, 1.1-1.4 mg/kg/day	CR, *n* = 1, aged 3 years	Difficulties falling asleep	∼27 months	BMI: decrease from 33.0 to 21.8 kg/m^2^ (-11.2) Vital functions: slightly lowered BP and HR Appetite: profound appetite suppression Weight loss, body composition, metabolic, others: N.R.
30.	Brandt et al, 2020 (25)	Heterozygous *MC4R*, homozygous *LEPR*	Methylphenidate, 20 mg/day	CS, n = 5, aged 2.8-14.9 years	Disordered sleep, nervousness, hyperactivity, and increased tics, increase liver enzymes in n = 1, increased diastolic pressure in n = 1 (within normal ranges), increase of dosage of antihypertensive treatment in n = 1	12 months	BMI: BMI -0.7 ± 0.9 kg/m^2^ (range, +0.7 to −1.9), BMI SDS -0.32 ± 0.20 (range, -0.03 to -0.64), %BMIP95 -6.6 ± 7.8% (range, +5 to -17), BMI-SDS velocity decreased from +0.17 ± 0.22 to -0.30 ± 0.20 Appetite: decrease in appetite in VAS (range, 1-10) from 8.3 ± 1.6 to 3.5 ± 2.4, decrease in “food responsiveness” and “enjoyment of food Weight, body composition, vital functions, metabolic, others: N.R.
31.	Van Schaik et al, 2022 (26)	*GNAS* (n = 1), *BBS1* (n = 1), *LEPR* (n = 2), PWL (n = 1), *mUPD14* (n = 1)	Dextroamphetamine, max 0.5 mg/kg/day (max of 40 mg/day)	Cohort, n = 6, aged 2.7-18.0 years	Hypertension in n = 2, difficulties falling asleep, behavior problems	23.7 ± 12.7 months	PHP-1a: increase BMI SDS, -0.5% FM *LEPR* patient 1: stabilization BMI SDS, + 1.7% FM PWL: stabilization BMI SDS mUPD14: decrease BMI SDS, −4% FM *BBS1*/*LEPR* patient 2: hypertension, resulting in stopping of treatment Appetite, metabolic, others: N.R.
32.	Hainerová et al, 2011 (83)	Homozygous *MC4R*	Sibutramine, 10 mg/day	CR, n = 1, aged 18 years	None	1 year	Weight: stabilization with +1.4 kg (+0.8%) BMI: from 55 kg/m^2^ to 55.5 kg/m^2^ (+0.5) Vital functions: unchanged Body composition: FM -2.4 kg (-2.8%) Metabolic: improved lipids, HOMA-IR, liver enzymes Appetite: improved VAS hunger score Others: +10.8 kg weight after cessation
33.	Kor et al, 2017 (84)	Homozygous *LEPR*	Fluvoxamine, 2 mg/kg/day	CR, n = 1, aged 12 months	None	3 months	Weight: -1 kg (-4.3% BMI: from 36.8 kg/m^2^ to 32.7 kg/m^2^ (-4.1) Appetite: severe appetite reduction Body composition, vital functions, metabolic, others: N.R.
34.	Stalman et al, 2015 (85)	mUPD14	Somatropin SC, patient A 0.48–0.53 mg/m^2^/day, patient B 0.34 mg/m^2^/day	CS, n = 2, aged 6.9 years (patient A) and 9.3 years (patient B)	None	2 years	Patient A: BMI from 17.4 kg/m^2^ (+1.4 SD) to 16.3 kg/m^2^ (+0.3 SD), FM +0.9 kg (-6.1%), LBM +3.9 kg, improved muscle strength Patient B: BMI from 21.8 kg/m^2^ (+2.4 SD) to 25.6 kg/m^2^ (+2.6 SD), FM +9.5 kg (+1.7%), LBM +7.4 kg, improved muscle strength
35.	Hebach et al, 2021 (86)	*MAGEL2*	Somatropin SC, average 0.23 ± 0.09 mg/kg bodyweight/week	Cohort, n = 14, mean age 2.7 years (range, 0.4-8.0)	Worsening OSA in n = 1, worsening scoliosis/kyphosis in n = 2	Mean 3.07 years (range, 0.38-15.78), evaluation treatment at 6 months	Weight SDS: -0.6 SD ± 1.1 to -1.0 SD ± 1.4 (n = 12) Weight-for-height SDS: 1.0 SD to 0.2 SD (n = 12) BMI SDS: + 1.3SD ± 0.9 to +0.7SD ± 1.1 (n = 7) Others: subjectively reported increase in muscle strength and endurance, improved cognitive and social skills (n = 6) and improved motor development (n = 5)
36.	Juriaans et al, 2023 (87)	mUPD14	Somatropin SC, 0.5 mg/m^2^/day and 1.0 mg/m^2^/day (∼0.035 mg/kg/day)	Cohort, n = 13, median age 7.3 (IQR 5.0 to 8.4)	None	5 years	BMI SDS: unchanged on group level (mean BMI SDS at baseline -0.69 (95% CI, -2.26 to 2.23) Body composition: At baseline: FM% SDS 2.65 (95% CI, 2.20; 3.09), LBM SDS -2.55 (95%CI -3.55; 1.56) After 1 year: FM% SDS 2.31 (95% CI, 1.85 to 2.77), LBM SDS -1.90 (95% CI, -2.88; -0.92) After 5 years: FM% SDS 2.20 (95%CI 1.70; 2.77), LBM SDS -0.33 (95%CI -1.37; 0.71) Other: REE unchanged Weight, vital functions, metabolic, appetite: N.R.
37.	Krude et al, 2003 (88)	Biallelic *POMC*	Intranasal ACTH4-10, 1-5 mg/d	CS, n = 2, children (age N.R.)	N.R.	3 months	Weight: unchanged BMI: unchanged Body composition: unchanged Vital functions, metabolic: N.R. Appetite: unchanged Others: REE% unchanged
38.	Shoemaker et al, 2023 (89)	*GNAS*	Theophylline oral, individualized dose	Placebo-controlled RTC, n = 11, mean age 17.2 ± 12.9 years (ranges 9-55)	Transient nausea, vomiting	30 months	BMI: baseline BMI%P95 133.5% ± 19.9 → at last visit BMI%P95 124.7% ± 18.6 (*P* = 0.06), 36% of patients decrease of >20% in BMI%P95 Weight, vital functions, body composition, metabolic, appetite, others: N.R.
39.	Ozsu et al, 2023 (90)	Biallelic *LEP*	Mibavademab SC, individualized dose	CR, n = 1, aged 5 years	No safety signals	60 weeks	Weight: −61.0 kg (−67.8%) BMI: −4.7 BMI-SDS Metabolic: improved glucose tolerance, insulin, and liver parameters Others: resolution of OSA Vital functions, body composition, appetite: N.R.

Abbreviations: BBS, Bardet-Biedl syndrome; BDNF, brain-derived neutrotrophic factor; BMI%P95, %BMI of P95th; BMI, body mass index; BP, blood pressure; CR, case report; CS, case series; DBP, diastolic blood pressure; DM2, type 2 diabetes; FFM, fat-free mass; FM, fat mass; GI, gastrointestinal; GNAS, guanine nucleotide-binding protein, alpha-stimulating activity; HOMA-IR, Homeostatic Model Assessment for Insulin Resistance; HR, heart rate; LBM, lean body mass; LEP, leptin gene; LEPR, leptin receptor; MAGEL2, MAGE family member L2; MC4R, melanocortin-4 receptor; MKS1, MKS transition zone complex subunit 1; mUPD14, maternal uniparental disomy for chromosome 14; N.R., not reported; NRCT, non-randomized controlled trial; OSA, obstructive sleep apnea; POMC, pro-opiomelanocortin; PWL, Prader-Willi like; REE, resting energy expenditure; SBP, systolic blood pressure; SC, subcutaneously; SDS, standard deviation score; VAS, visual analogue scale.

### Lipase Inhibitor

#### Mechanism of action

Lipase inhibitors have been on the market the longest, with orlistat being the most well-known lipase inhibitor. In its natural form, lipoprotein lipase is a multifunctional enzyme responsible for breaking down lipids, hydrolyzing fats into fatty acids and glycerol, and enhancing fat absorption by extracting triglycerides from the circulation (91). Orlistat is a derivative of endogenous lipstatin, which is produced by a *Streptomyces toxytricini*. It functions in the intestinal tract by reversibly inhibiting lipases, forming covalent bonds with active serine sites of the gastric and pancreatic lipases, leading to their inactivation. This process blocks fat hydrolysis, resulting in reduced fat absorption by roughly 30% when using 3 times daily 120 mg (91).

#### Effects in common obesity

Several studies have shown a modest effects of orlistat on weight loss in patients with common obesity. A recent network meta-analysis, including 49 810 patients, reported a decrease during orlistat treatment of -3.16% (95% CI, -3.53 to -2.78) of body weight at follow-up (duration treatment between 12 and 104 weeks) compared to lifestyle modification alone (92). Another large meta-analysis in 10,435 patients with multifactorial obesity treated with orlistat, demonstrated a decrease of -3.07 kg (95% CI, -3.76 to -2.37) after ≥12 months of treatment (93). A meta-analysis analyzing the effects of orlistat in children with obesity showed no significant effect of orlistat on body weight, BMI, or metabolic parameters (94). Besides this modest decrease in weight, a large proportion of patients, ranging between 6.4% and 48.5%, has been reported as discontinuing orlistat treatment because of a lack of therapeutic response and/or side effects (92, 93, 95, 96). These side effects are predominantly related to gastrointestinal symptoms due to fat malabsorption, such as abdominal pain, steatorrhea, fecal incontinence, flatulence, or diarrhea (97). Additionally, the reduced fat absorption can lead to decreased absorption of fat-soluble vitamins, such as vitamins A, D, and E.

#### Effects in genetic obesity

One case report describes the effect of several antiobesity therapies in a 17-year-old child with a heterozygous pathogenic *MC4R* variant. Lifestyle changes, metformin, and orlistat treatment did not lead to reduction of body weight in this patient. Besides this case report, no other studies have been published about the effects of orlistat in patients with genetic obesity (61).

### Short-term Phentermine

#### Mechanism of action

The centrally acting noradrenergic drug phentermine has been approved in the United States for short-term use (≤12 weeks) since the 1950s because of the lack of long-term data. It is thought to mainly act on the reward system, specifically the nucleus accumbens, by enhancing the release of norepinephrine, and to a lesser extent serotonin and dopamine (98, 99). These increased neurotransmitter concentrations mediate the reward value of food and, consequently, affect appetite (100). However, most studies examining mechanism of action have been done in rodents and not in humans.

#### Effects in common obesity

The longest RCT of 36 weeks of phentermine treatment in adults with obesity revealed a placebo-subtracted weight loss of -7.4 kg and -8.2 kg when administered continuously or intermittently (every 4 weeks alternating between phentermine and placebo), respectively (101). Phentermine treatment induced a significantly greater reduction of cravings for fats and sweets, compared to the placebo-treated group (102). The most frequently reported side effects of phentermine include dry mouth, insomnia, dizziness, palpitations, constipation, and psychiatric adverse effects such as agitation, irritability, and anxiety (101, 103).

#### Effects in genetic obesity

The only study reporting on phentermine treatment in patients with genetic obesity is a recent case series in 6 patients with heterozygous pathogenic *MC4R* variant (67). This showed a significant weight decrease of -12.7 ± 2.3 kg (-15.5% ± 2.9) after 6 months of treatment, which was similar to the control group (-11.3 ± 0.9 kg of body weight, -13.6% ± 1.1). Additionally, improvements of percentage fat mass and muscle mass, several glucose indices, and leptin were observed, although not significantly (67). Similar side effects as in patients with common obesity were observed.

### Phentermine-topiramate

#### Mechanism of action

Topiramate as monotherapy has weight-reducing potential, as was shown in an uncontrolled, prospective study in which weight loss was observed in adults with epilepsy treated with topiramate. The amount of weight loss in this study strongly correlated with a reduced caloric intake, implying topiramate-induced appetite inhibition (104). Topiramate is a carbonic anhydrase inhibitor and glutamate receptor antagonist. When given in combination with phentermine, the effects on appetite suppression and increasing satiety seem to be enhanced (103). However, the exact mechanism of action of topiramate on appetite and weight loss is not fully understood yet.

#### Effects in common obesity

Two large RCTs in 1267 and 2487 adults with obesity or overweight combined with ≥2 obesity-related comorbidities have shown placebo-subtracted weight losses at maximum dose of -9.3% and -8.6% after 56 weeks of phentermine-topiramate treatment (105, 106). An extended RCT after 106 weeks of treatment reported a sustained effect (placebo-subtracted weight loss of -8.7%) (107). In all 3 RCTs, improvements in metabolic parameters were reported as well (105-107). This treatment has shown to be effective in an RCT including children aged ≥12 years with obesity as well: a -10.4% (95% CI, -13.9 to -6.9) decrease in BMI and improvement of lipids were observed after 56 weeks of treatment at maximum dose, compared to placebo (108). The most common side effects were constipation, paresthesia, dry mouth, dysgeusia, and psychiatric adverse events such as insomnia and anxiety (105-107).

#### Effects in genetic obesity

No literature is available about the effects of phentermine-topiramate treatment in patients with genetic obesity. The study of Salazar et al shows the potential of phentermine treatment as monotherapy in patients with *MC4R* variants (67). They reported a weight decrease of -12.7 ± 2.3 kg (-15.5% ± 2.9) after 6 months of treatment, which was similar to a control group of patients with obesity (67). Therefore, we postulate that the combination of phentermine and topiramate as obesity treatment might be even more effective than phentermine monotherapy.

### Naltrexone-bupropion

#### Mechanism of action

Naltrexone as monotherapy seems to be insufficient to induce weight loss, whrtrsd bupropion showed a modest placebo-subtracted weight loss of -2.77 kg, as shown in a meta-analysis (109, 110). However, the combination of the 2 yields greater potential (110). It targets the 2 most important pathways involved in regulating weight and appetite: the homeostatic leptin-melanocortin pathway snf the hedonic reward-related pathway (109). Bupropion is thought to act on the arcuate hypothalamic nucleus by stimulating the MC4R via the induction of secretion of α-MSH by POMC neurons. Naltrexone on its turn blocks the opioid receptor-mediated POMC autoinhibitory loop and hereby enhances the bupropion-induced POMC activation (109). In addition, both naltrexone and bupropion affect the reward system via increasing the catecholamine's dopamine and norepinephrine in the nucleus accumbens, which is one of the key nuclei of the mesolimbic reward system (109). Overall, this leads to an anorexigenic effect, resulting in weight loss.

#### Effects in common obesity

A large RCT, including 1496 adults with overweight or obesity, revealed a -6.4% weight loss after 56 weeks of treatment vs -1.2% in the placebo-treated group (111). Additionally, improvements in waist circumference, cardiometabolic risk factors, weight-related quality of life, and control of eating were observed (111). A subsequent RCT in 793 adults with obesity revealed the additive effect of intensive group behavior modification next to naltrexone-bupropion treatment (-9.3 ± 0.4% vs -5.1 ± 0.6% in the placebo-treated group) (112). Frequently reported side effects were nausea, constipation, headache, insomnia, dry mouth, and dizziness (111). Clinicians should be aware of possible psychiatric adverse effects, such as anxiety, depression, and sleep disorders (113). No literature is available about the effects of naltrexone-bupropion in children with obesity; consequently, naltrexone-bupropion is not approved by the FDA and EMA for obesity treatment in children.

#### Effects in genetic obesity

Until now, 2 studies report on the effects of naltrexone-bupropion on patients with genetic obesity disorders. The largest real-world study includes 11 patients with molecularly confirmed genetic obesity. A weight loss of -5.2% ± 5.8, decrease in fat mass of -3.9% ± 2.8, improved obesity-related comorbidities, and improved self-reported appetite in 90.9% of the patients was observed. Similar results were seen in patients with a clinical phenotype of a genetic obesity disorder, but without definite diagnosis yet (114). The other study reporting on the effects of naltrexone-bupropion in genetic obesity, is a case report about 1 patient with a heterozygous pathogenic *MC4R* variant and an extensive medical history, including bariatric surgery and liraglutide treatment, with insufficient effect. In this case report, an impressive weight-reducing effect of -48.9 kg (-26.7%), of which -39.9 kg (-38.3%) fat mass, was observed together with an improvement of hyperphagia and quality of life after 17 months of treatment (22). This patient experienced nausea at higher doses, which is also a frequently reported side effect in patients with common obesity. More studies are needed to investigate the effectiveness of naltrexone-bupropion on obesity and hyperphagia in patients with genetic obesity.

### Liraglutide and Semaglutide

#### Mechanism of action

Currently, short-acting 3.0 mg liraglutide and long-acting 2.4 mg semaglutide, both GLP-1 analogues, are FDA and EMA approved for regular obesity management. These are both acylated human GLP-1 analogues and have a 94% to 97% homology regarding amino acid sequence to endogenous human GLP-1. Both liraglutide and semaglutide exhibit their function by mimicking the effects of the endogenous GLP-1, which is mainly released by intestinal L cells upon food intake (115). Upon binding, the GLP-1 receptor is activated. This results in increased glucose-dependent release of insulin, inhibition of glucose-dependent release of glucagon, and delayed gastric emptying. Additionally, reduced appetite is achieved via affecting hypothalamic neuronal pathways, including the neurons involved in the homeostatic leptin-melanocortin pathway, as well as the hedonic-related neurons (115, 116). As GLP-1 receptors are widely expressed across the body, such as in the gastrointestinal tract, brain, heart, vasculature, immune system, and kidneys, the effects of GLP-1 analogues on weight and appetite seem to be pleiotropic (115). This was shown in several studies using GLP-1 analogues, which showed protective cardiovascular effects, anti-inflammatory effects, and antiaddiction effects (117-123). Animal studies have shown that these protective cardiovascular effects may be due to reduced inflammation, improved endothelial and left ventricular function, enhanced plaque stability, and decreased platelet aggregation (123).

#### Effects in common obesity

The first RCT in adults with obesity treated with once daily subcutaneous injection of 3.0 mg liraglutide showed a mean reduction of -8.0% ± 6.7 of their body weight after 56 weeks of treatment, whereas the placebo group had lost a mean of -2.6% ± 5.7 (124). Subsequently, an RCT reporting the long-term effects of liraglutide has shown a mean weight reduction of -6.1% ± 7.3 after 160 weeks of treatment, which was significantly higher than the placebo group (-1.9% ± 6.3) (125). Also in children with obesity, these beneficial effects were observed. Compared to placebo, an estimated treatment difference due to liraglutide in BMI SD score of −0.22 (95% CI, -0.37 to -0.08) and relative BMI change of -4.64% (95% CI, -7.14 to -2.14) were reported in an RCT (126). Semaglutide 2.4 mg treatment led to large weight reductions in both children and adults with obesity. A mean reduction of -14.9% of body weight in adults with obesity, compared to -2.4% in the placebo group, was reported after 68 weeks of treatment in an RCT (127). This effect sustained after 2 years of treatment (128). In these RCTs, semaglutide was able to successfully suppress appetite leading to improved control of eating and reduced food cravings at both first and second year of treatment (129, 130). An RCT including adolescents with obesity reported a similar weight reduction of -16.1% in the semaglutide treated group compared to +0.6% in the placebo group. Additionally, improvements in waist circumference and cardiometabolic risk factors in both adults and children were observed (118, 131). Semaglutide has also been proven to have cardiometabolic protecting potential (117, 119). Both liraglutide 3.0 mg and semaglutide 2.4 mg were shown to have an additive effect, next to lifestyle interventions such as very low-calorie diet or intensive behavioral therapy, on weight loss (132, 133). The most commonly reported adverse events of GLP-1 analogues are largely gastrointestinal in nature, such as nausea, vomiting, constipation, and diarrhea. Acute pancreatitis has been reported as a severe but very rare adverse effect during GLP-1 analogue treatment; however, the exact mechanism behind this remains unknown (134, 135).

#### Effects in genetic obesity

GLP-1 analogues are the most studied nontargeted antiobesity agents in patients with genetic obesity. Recently, a real-world study in 30 adults with AS treated with GLP-1 analogues showed a mean body weight decrease of -5.4 kg (-6%) ± 1.7, improved HbA1c, lipid profile, and ALT, and improved satiety using a visual analogue score after 6 months of treatment (64). Another observational study reported a -4.7% (interquartile range, -6.0 to -1.5%) decrease in body weight, -0.9% ± 2.7 decrease in fat mass, improvements of obesity-related comorbidities, and improved self-reported appetite in 83.3% of 18 adults with genetic obesity after a median of 4 months of treatment. Similar results were seen in patients highly suspected for genetic obesity, but without definite diagnosis (114). Another observational study, including 14 adults with heterozygous MC4R deficiency, showed a weight loss of -5.7% ± 1.4 after 16 weeks of liraglutide treatment. Body composition and cardiometabolic factors, such as fat mass, waist circumference, and fasting glucose, also improved (23). Other studies are smaller case series, such as the case series published by Welling et al reporting the effects of GLP-1 analogue treatment in 4 adults with 16p11.2 deletion syndrome or MC4R deficiency. They reported weight losses during GLP-1 analogue treatment ranging between -6.1% to -27.6% (treatment duration ranging between 43 weeks and 12 years). Three adults also reported reduced hyperphagia and improved satiety during GLP-1 analogue treatment (21). Also in children with various types of genetic obesity, such as heterozygous and homozygous MC4R deficiency and 16p11.2 deletion syndrome, beneficial effects of liraglutide on body weight, cardiometabolic factors, and appetite regulation are reported (60, 61, 63, 65, 66). The potential of GLP-1 analogues in BBS was shown in a case report of a patient with BBS type 10 who lost 29.3% of body weight and improved glycemic control after 40 weeks of treatment (62). Similar side effects with respect to type and frequency were observed as in patients with common obesity.

### Tirzepatide

#### Mechanism of action

Recently, tirzepatide has been approved by the FDA and EMA for obesity treatment as the first long-acting dual GIP and GLP-1 receptor agonist.

GIP serves as the main incretin hormone, which triggers insulin secretion from pancreatic β cells in response to elevated glycose levels during meals among healthy individuals. It also regulates glucagon release during hypoglycemic and hyperglycemic periods (136). Activation of GIP, therefore, leads to improved glycemic control and insulin sensitivity. It also enhances clearing of dietary triglycerides by adipocytes and lipid-buffering capacity of the white adipose tissue (136). Additionally, GIP appears to act centrally on different regions in the hypothalamus leading to reduced food intake and weight loss (136). In summary, activation of both GIP and GLP-1 receptors works synergistically via enhancing GLP-1-mediated weight loss.

#### Effects in common obesity

A -20.9% (95% CI, -21.8 to -19.9) weight loss was observed in an RCT including 2539 adults with obesity after 72 weeks of 15 mg tirzepatide treatment, compared to -3.1% (95% CI, -4.3 to -1.9) in the placebo-treated group (137). Improvements in waist circumference, glycemic control, and other cardiometabolic parameters were also observed (137). Frequently reported side effects of tirzepatide were nausea, diarrhea, constipation, dyspepsia, vomiting, and headache (137). Hypoglycemic periods may occur when concomitantly using sulphonylurea, insulin, or metformin (138).

#### Effects in genetic obesity

One case report is available that reports on tirzepatide treatment in a 33-year-old female, in whom genetic testing revealed a heterozygous variant of unknown significance in the *MC4R* gene and *Meckel Syndrome Type 1 protein* gene. Tirzepatide treatment (dosing unknown) led to a -28.0 kg (-20%) decrease of excess body weight during 3 months of treatment (139).

## Off-label Use of Other Pharmacological Agents

Several pharmacological agents have been tried and used for genetic obesity treatment. All studies reporting the effects of these nontargeted off-label pharmacological agents in patients with genetic obesity disorders are summarized in Table 4. Most studies reporting on the effects of the off-label pharmacological agents in patients with genetic obesity are observational in nature, including case reports, case series, and small cohort studies.

### Metreleptin

Leptin analogues are currently used off-label for obesity treatment in patients with hypoleptinism due to congenital leptin deficiency (CLD). Leptin analogues mimic the physiological effects of the endogenous leptin, and upon binding with the leptin receptor, restore the defective leptin-melanocortin pathway in the hypothalamus and other neuronal populations of the dopaminergic reward system (140). Functional magnetic resonance imaging studies in a patient with CLD showed reestablished brain insulin sensitivity, particularly of the hypothalamus, after 1 year of metreleptin treatment (141). Additionally, it is able to induce long-term functional brain activation changes, assessed using functional magnetic resonance imaging, in brain areas involved in homeostatic weight regulation (ie, the hypothalamus), but also in reward-related brain areas, such as the striatum, orbitofrontal cortex, and amygdala (142). Probably, changes in gut hormones, such as reduction of ghrelin and increase in peptide YY, contribute to the improved satiety during metreleptin treatment in patients with CLD (76).

The first RCT in patients with common obesity showed variable weight losses during metreleptin treatment, with the greatest weight loss achieved in the high-dose group (143). Subsequent RCTs in different patient populations with obesity did not reveal a role of leptin replacement therapy in inducing weight loss (144-146). This might be due to patients with common obesity not being leptin deficient, but on the contrary having high leptin levels and being leptin resistant. As studies have shown minimal impact of leptin replacement therapy on common obesity, leptin analogues have currently no role in treatment of common obesity. The astounding effects of leptin replacement therapy for CLD were first reported in case series, including children with CLD. These showed beneficial effects on weight (range, -3.4% to -24.8%), BMI (range, -12.4% to -44.2%), fat mass (range, -4.1% to -20.8%), metabolic parameters, and appetite after 6 to 48 months of treatment (68-70, 74). Metreleptin treatment was also shown to be effective in a young patient aged 3 years with biologically inactive leptin and 2 children aged 2 and 14 years with antagonistic leptin variants (75, 81). A larger case series of 9 children with CLD, published in a conference paper, showed a reduction of -1.4 SD ± 0.8 of BMI-SD score, increased IGF-1 levels, and accelerated growth in children aged ≤12 years (77). Additionally, in 7 children with CLD, improved indices of physical activity and psychological wellbeing were observed during 4 months of metreleptin treatment (79). Case series, including adults with CLD, demonstrated similar results on the short-term (weight loss ranging between -11.9% to -13.9% after 19 weeks of treatment) and long-term (weight loss ranging between -32.8% to -55.3% after 18 to 72 months of treatment) (71-73, 80). Next to being compliant to daily metreleptin treatment, it is important to also adhere to a healthy lifestyle to maximize the effects of metreleptin treatment on weight, metabolic parameters and eating behaviors (78). A possible side effect of leptin analogue treatment is the development of neutralizing antibodies, resulting in partial or complete loss of efficacy (147). No other side effects in patients with CLD treated with leptin analogues have been reported yet, including studies with a follow-up of up to 7 years. This should be further assessed in future studies since the number of metreleptin-treated patients with CLD was small. In patients with lipodystrophy treated with leptin analogue treatment, nausea and hypoglycemia, specifically in patients with T2D as comorbidity, have been reported as very common side effect (148). These hypoglycemia episodes were generally mild in nature and could be managed by food intake or adjusting of antidiabetic medications.

### Metformin

Until recently, it was thought that reducing hepatic gluconeogenesis and pancreatic insulin production were the main drivers of weight loss during treatment with metformin, a biguanide derivate. However, metformin effects seem to be more pleiotropic with interesting central signaling effects seen in animal studies. Examples are improved leptin sensitivity plausibly via hypothalamic leptin receptor expression in rodents and inhibition of adrenocorticotropic hormone induced MC2R and MC3R activation as well as α-MSH-induced MC3R activity (149, 150). Additionally, it was able to upregulate circulating levels of growth differentiation factor 15, which can act as a mediator with positive effects on energy intake and expenditure (151). Very recently, it was discovered that metformin administration leads to increasing levels of the metabolite N-lactoyl phenylalanine, with appetite suppression as result (152). Potent anti-inflammatory effects of metformin have also been described, which can aid in treatment of obesity of cardiovascular disease as well (153). This weight loss potential was shown in a meta-analysis investigating the effect of metformin on weight in different adult populations with obesity, which showed an overall modest decrease in BMI: a weighted mean difference of -0.98 kg/m^2^ (95% CI, -1.25 to -0.72). The largest impact of metformin treatment was observed in patients with common obesity (decrease in BMI of -1.31 with 95% CI of -2.07 to -0.54), compared to patients with obesity combined with metabolic diseases such as T2D, polycystic ovary syndrome, insulin resistance, and/or impaired fasting glucose (154). In children, the effects of ≥6 months of metformin on absolute weight and BMI are smaller (ΔBMI ranging between -2.4 and 2.2 kg/m^2^) compared to adults (ΔBMI ranging between -1.4 and -0.4 kg/m^2^), possibly due to differences in therapy compliance, daily dosage, and insulin status (155). However, use of absolute BMI as outcome is not preferable in children because BMI is sex- and age-dependent during childhood. Therefore, the smaller effects on BMI observed in this systematic review may possibly be considered as beneficial as in adults with obesity. One case report in a child with homozygous POMC deficiency demonstrated a BMI decline from 34.9 to 32.9 kg/m^2^ during approximately 3 years of metformin treatment. Unfortunately, no effects on appetite were described (82). On the contrary, a case report in a child with homozygous MC4R deficiency did not reveal an impact of metformin treatment on appetite and weight (63). Unfortunately, no larger studies have been published on the effect of metformin in patients with genetic obesity. As studies with metformin have shown appetite-reducing potential via modulating of the abovementioned central hypothalamic signaling pathways, further research is needed to investigate the effectiveness of metformin on appetite and weight in patients with genetic obesity and impaired appetite regulation. The most common side effects of metformin are mild, transient, and mainly gastrointestinal of nature. Most often nausea, vomiting, diarrhea, and abdominal pain are reported. A rare side effect of metformin is lactic acidosis, which can arise due to overdose or in contraindicated conditions (156).

### Central Nervous System Stimulants

As with phentermine, agents such as methylphenidate and dextroamphetamine are thought to impact both the hedonic dopaminergic system as well as the homeostatic leptin-melanocortin pathway in the hypothalamus (157, 158). Methylphenidate inhibits the dopamine and norepinephrine (NE) transporters in the presynaptic neurons, leading to decreased dopamine and NE reuptake and, consequently, increased dopamine and NE concentration in the synaptic clefts. Additionally, it functions as a weak serotonin-1A receptor agonist, which has a positive cumulative impact on the levels of dopamine (159, 160). Next to inhibiting reuptake of neurotransmitter via inhibition of the dopamine and NE transporters, dextroamphetamine also promotes release of dopamine by inhibiting of the vesicular monoamine transporter 2 and via reverse transport by the dopamine transporter (DAT). Additionally, amphetamines inhibit monoamine oxidase activity, resulting in decreased breakdown of cytosolic monoamines (159). These increased dopamine and NE results in its characteristic stimulant effect within the central nervous system and mediating the value of reward regarding food, this change in dopamine concentration impacts appetite signaling (100, 157, 158). Multiple studies have reported on the effect of dextroamphetamine on weight and appetite in children with acquired hypothalamic obesity; however, less is described in patients with genetic obesity (26, 161-163). One case report described the use of methylphenidate for 37 months as obesity treatment in a 3-year-old patient with heterozygous MC4R deficiency, which had resulted in a -11.2 kg/m^2^ decrease in BMI and improvement of hyperphagia (24). A case series including 2 children with heterozygous MC4R deficiency and 3 children with biallelic LEPR deficiency treated with methylphenidate for 12 months, showed beneficial effects as well: decrease in BMI of -0.7 ± 0.9 kg/m^2^ (range, +0.7 to -1.9), decrease in %BMIP95 of -6.6 ± 7.8% (range, +5 to -7) and decreased appetite, food responsiveness, and enjoyment of food (25). A small cohort study, including 19 children with hypothalamic obesity, revealed similar beneficial results on BMI (BMI SD score from 3.58 ± 0.85 [range, 2.46-5.05] to 3.18 ± 1.44 [0.95-5.68] at last moment of follow-up) and appetite. When zooming in on the subgroup of patients with genetic obesity, it seemed that the effects were less pronounced in this subgroup of patients compared to the patients with acquired hypothalamic obesity (26). Most common reported side effects in all studies were difficulties falling asleep and behavioral problems, such as nervousness, hyperactivity, and anger (24-26). Additionally, clinicians should be aware of the possible effect of both dextroamphetamine and methylphenidate on blood pressure as increases in blood pressure have been reported as have potential other long-term adverse effects (25, 26).

### Sibutramine

Sibutramine is also a centrally acting drug which selectively inhibits the neuronal reuptake of serotonin and norepinephrine, and to a lesser extent, dopamine within the hypothalamus. Weight loss is then achieved via decreasing of appetite, improving of satiety, and possible affecting energy expenditure as well (164). It was withdrawn from the market as there were cardiovascular safety concerns, mainly for long-term sibutramine treated patients with preexisting cardiovascular conditions (165). There is, however, 1 case report that describes the effect of sibutramine treatment in an adult with homozygous MC4R deficiency with progressive weight gain (approximately 20 kg/year). Sibutramine treatment resulted in weight stabilization (+1.4 kg in 12 months of treatment), improved hyperphagia, and improved lipids, Homeostatic Model Assessment for Insulin Resistance, and liver enzymes. Weight (re)gain after cessation of treatment was +10 kg in 12 months (83).

### Fluvoxamine

As fluvoxamine is a selective serotonin reuptake inhibitors, its effect on weight is mediated via modulating of serotonin levels in the hypothalamus, leading to appetite suppression (166). An RCT from 1993 demonstrated a mean -3.5 kg weight loss after 12 weeks of treatment, which was not significant compared to the placebo-treated group (mean −2.8 kg) (167). Afterwards, its use in treatment of binge eating disorders was investigated which showed minimal effects as well (168). Commonly reported side effects are mainly gastrointestinal, such as nausea, but also somnolence and asthenia (169). The impact of fluvoxamine treatment in a 12-month-old girl with biallelic LEPR deficiency has been reported. In the 4 months prior to fluvoxamine treatment, the child gained +6.9 kg of body weight, whereas during 3 months of fluvoxamine treatment she lost 1 kg of body weight and her appetite was adequately suppressed (84). The child did not experience any side effects.

### Growth Hormone

The effects of GH treatment have been studied extensively in children with Prader-Willi syndrome and GH deficiency. A recent meta-analysis showed improvements in BMI SD scores and body composition (170). One case series describes the effects of GH in 2 children with maternal uniparental disomy of chromosome 14 (Temple syndrome), which led in both patients to increased growth and subjectively reported increased muscle strength after 2 years of treatment. Interestingly, decreases in BMI (-1.1 kg/m^2^, -1.1 SD) and fat mass (-6.1%) in the patient with normal BMI at baseline were observed, whereas increases in BMI (+3.8 kg/m^2^, + 0.2 SD) and fat mass (+1.7%) were observed in the other patient with obesity at baseline (85). A larger observational study, including 13 children with Temple syndrome, reported improved body composition, such as significant decreases in fat mass percentage SD score and lean body mass SD score, after 5 years of growth hormone treatment (87). No side effects were observed in both studies. Children with Schaaf-Yang syndrome also have been treated with GH treatment with a decreasing BMI SD score as result (86). It should, however, be noted that in this study mean BMI SD score (+1.3 ± 0.9) was below the obesity definition at start of treatment. Research evaluating the effects of continuing growth hormone treatment from adult age onwards and the effects of other AOMs in these patients is needed.

### Intranasal ACTH 4-10

Like setmelanotide, ACTH 4-10 exerts its function via stimulation of the MC4R, located in the leptin-melanocortin pathway. It was first studied in humans with normal weight, with significant differences in fat mass (-1.68 kg), body weight (-0.79 kg), plasma leptin (-24%), and insulin (-20%) during 6 weeks of twice daily ACTH 4-10 as result (171). A study in which 2 patients with biallelic POMC deficiency were treated with intranasal ACTH 4-10 followed. Remarkably, this did not affect weight, body composition, and basal metabolic rate (88). The postulated reason behind this lack of effect was the 1000 times less affinity of intranasal ACTH 4-10 at the M4CR, compared to endogenous α-MSH.

### Theophylline

Theophylline is a nonselective phosphodiesterase inhibitor with smooth muscle relaxant, bronchial dilating, cardiac, and central nervous system stimulant properties (172). Hence, it is used for treatment of obstructive respiratory diseases such as asthma. In patients with maternally inherited inactivating *GNAS* variants, resulting in tissue-specific silencing of paternal expression, the G-protein coupled receptors, including those within the leptin-melanocortin pathway, dysfunction resulting in low cyclic adenosine monophosphate (cAMP) levels (173). This dysfunction leads to the phenotype of genetic obesity and multihormone resistance (174). It was hypothesized that these cAMP levels were low, and not completely absent, in patients with *GNAS* variants. As cAMP is rapidly degraded by phosphodiesterase inhibitor, theophylline could inhibit this breakdown of cAMP leading to increased G-protein coupled receptor signaling. Preliminary results of a phase 2 RCT, including 12 children and adults, were recently published in a conference paper, demonstrating a borderline significant decrease of BMI%P95 from 133.5% ± 19.9 to 124.7% ± 18.6 (*P* = 0.06). In 36% of patients a >20% decrease in BMI%P95 was observed. The reported adverse events were nausea and vomiting (89). The results of the completed phase 2 RCT should be awaited to draw final conclusions about theophylline treatment in patients with maternally inherited inactivating *GNAS* variants.

## Promising Antiobesity Therapies in Future

Currently, a great deal of research is being performed on new investigational agents for treating obesity (175). We have summarized the most promising ones here.

### Dual and Triple Agonists

It was postulated that dual agonists probably have larger weight-reducing potential because of synergistically functioning of multiple gut hormones, resulting in enhanced effects on appetite and energy expenditure. Indeed, the first dual GIP and GLP-1 receptor agonist to be EMA and FDA approved, tirzepatide, showed significant larger weight losses compared to GLP-1 analogues (137). Other dual agonists, including combinations of GLP-1/glucagon, amylin/calcitonin or amylin/GLP-1, are being investigated in clinical trials now (176-179). These agents may affect hypothalamic signaling as well, as shown in a recent study in mice with obesity treated with cotadutide, a GLP-1/glucagon analogue (180). On top of that, combinations with 3 different gut hormones, such as GLP-1/GIP/glucagon triagonists or GLP-1/oxyntomodulin/peptide-YY triple combination, have been developed and are also being investigated in clinical trials. Recently, an RCT reported on the remarkable dose-dependent effects of retatrutide, a GLP-1/GIP/glucagon triagonist, after 48 weeks of treatment. This revealed in 100% of the treated patients a weight loss of at least 5%, and mean weight loss of -8.7% in the 1-mg group to -24.2% in the 12-mg group, compared to -2.1% in the placebo group. Additionally, a significant decreased waist circumference, improved cardiometabolic measures, such as blood pressure, glycemic indices, and lipids, and normalization of prediabetes in 72% of patients were observed (181).

### Activin Type II Receptor Blocker (Bimagrumab)

One of the key players of regulating skeletal muscle mass is the activin type-IIB receptor, which has several natural ligands like myostatin, activin, and growth differentiation factor 11. Bimagrumab, a monoclonal anti-ActRII antibody, blocks this activin type IIB receptor. Blocking this receptor leads to inhibition of the process of negatively regulating skeletal muscle growth by natural ligands, resulting in muscle hypertrophy and enhanced recuperation after muscle wasting conditions (182, 183). Additionally, activation of brown adipogenesis and thermogenesis was observed in a mouse study (184). Bimagrumab was shown to be effective in an RCT including adults with insulin resistance, demonstrating a -7.9% decrease in fat mass, +2.7% increase in lean mass, and improved HbA1c and insulin sensitivity after 10 weeks of bimagrumab treatment (185). A phase 2 RCT in patients with obesity and T2D revealed similar results after 48 weeks of treatment: a decrease in fat mass of -20.5% (-7.5 kg, 80% CI, -8.3 to -6.6), increase in lean mass of +3.6% (1.70 kg, 80% CI, 1.1 to 2.3 kg), and decrease in weight of -6.5% (-5.9 kg, 80% CI, -7.1 to -4.7). Interestingly, the effects on lean mass in men were observed to be greater. After 48 weeks of treatment, 17/26 (65.4%) patients achieved at least 5% of weight loss. Frequently reported side effects were mild diarrhea and muscle spasms (186). Currently, an ongoing trial is investigating the effect of the combination of mibavademab and tirzepatide (NCT06373146).

### Mibavademab

The monoclonal antibody, mibavademab, acts as a leptin analogue by binding to the LEPR, with similar potency and activity as leptin, resulting in restored signaling (187). A phase 1 RCT in which adults with common obesity or overweight together with low baseline leptin levels (<5 ng/mL) were treated with mibavademab, demonstrated a placebo-subtracted weight loss of -2.8 kg (-3.1%) and -2.4 kg (-3.1%) in a phase 1 RCT. Less effect was observed in 2 cohorts with higher baseline leptin levels (187). Efficacy of mibavademab was demonstrated in a 5-year old child with congenital leptin deficiency and neutralizing antibodies for metreleptin in a published conference paper, reporting a -61.0 kg (-67.8%) weight loss, -4.7 decrease in BMI SDS, improved glucose tolerance, insulinemia, and liver parameters, and resolution of obstructive sleep apnea (90).

### Gene Therapy

As genetic obesity disorders are caused by genetic defects, genome editing therapy seems to be a promising solution to repair genetic disease-causing mutations. CRISPR-based gene editing technologies can directly repair the defective genes, using for example viral vector delivery, but also via induced pluripotent stems cells (188). Using these methods, the energy homeostasis is expected to be restored by replacing the mutant gene by a functional recombinant copy in the appropriate cells. In *ob/ob* mice with congenital leptin deficiency, the mutated *leptin* gene in the preadipocytes and inguinal adipose tissues was successfully corrected using CRISPR-based gene editing (189). Another method using CRISPR-based gene editing is to upregulate the existing functional gene copy in haploinsufficient diseases. This was shown to be effective in *sim1* or *mc4r* haploinsufficient mice injected with this therapy in the hypothalamus (190). Currently, limitations include the requirement to develop labor-intensive specific sequences for each distinct genetic defect, the inability to apply this method to microdeletions or large deletions, and the inability for the gene therapy to reach specific tissues and target specific tissues only (188).

## Conclusion

In patients with a genetic obesity disorder, treatment with antiobesity pharmacotherapy, in addition to lifestyle interventions, is often required to treat their obesity and hyperphagia. Treatment aims in patients with genetic obesity disorders differ compared to patients with common obesity, as stabilizing body weight can be considered a successful outcome due to the otherwise progressive nature of (genetic) obesity. Additionally, reducing hyperphagia is a crucial treatment goal because it could lead to significant reductions in body weight and minimizes the negative impact of hyperphagia quality of life. Given the significant stigma surrounding obesity, particularly extreme obesity, is still present, it is crucial for clinicians to be nonstigmatizing during treatment and potential relapses, and to be compassionate and supportive regarding the hyperphagia. Considering that obesity in itself is a chronic, progressive, and relapsing disease and genetic obesity is in particular more therapy-resistant for many conventional treatments, a trial-and-error treatment approach appears to be most appropriate. In the event that a particular AOM fails to yield the desired effects, it may be feasible to opt for an alternative AOM or combine it with another AOM. In the last couple of years, great advances in pharmacotherapeutical options for obesity have been made that led to the availability of several AOMs. Currently, patients with specific genetic obesity disorders can be treated with targeted pharmacotherapy like leptin replacement therapy and setmelanotide. The availability of these targeted agents underscores the importance of genetic testing in patients suspected for genetic obesity disorders. Additionally, nontargeted AOMs, such as naltrexone-bupropion, GLP-1 analogues, or incretin-based dual agonists, are approved for obesity in general, including those with genetic obesity disorders. As a pharmacological treatment guideline for genetic obesity (except for congenital leptin deficiency), we propose initially starting treatment with approved nontargeted AOMs, such as incretin-based drugs, as these have relatively low costs and are widely available. In case of inadequate response after 3 to 4 months, treatment can be switched to other nontargeted AOMs. For patients aged 6 years and older with POMC deficiency, PCSK1 deficiency, LEPR deficiency, or BBS, MC4R agonists are approved which could be considered when available and reimbursed. The question remains which treatment is effective for whom. In addition, the long-term effects of all AOMs in genetic obesity need to be awaited. In the future, novel and innovative pharmacotherapeutical options, including combination therapies and gene therapy, will emerge, offering promising effects on body weight, hyperphagia, and, most importantly, quality of life.

## Abbreviations

AOM: antiobesity medication
AS: Alström syndrome
BBS: Bardet-Biedl syndrome
BMI: body mass index
CLD: congenital leptin deficiency
EMA: European Medicines Agency
FDA: Food and Drug Administration
GIP: glucose-dependent insulinotropic polypeptide
GLP-1: glucagon-like peptide-1
LEPR: leptin receptor
MC4R: melanocortin-4 receptor
MSC: melanocyte-stimulating hormone
NE: norepinephrine
PCSK1: proprotein convertase subtilisin and kexin type 1
POMC: pro-opiomelanocortin
PWS: Prader-Willi syndrome
RCT: nonrandomized controlled trial
T2D: type 2 diabetes
